# Progress of MXene-Based Materials in the Field of Rechargeable Batteries

**DOI:** 10.3390/ma18102386

**Published:** 2025-05-20

**Authors:** Jianfei Gao, Jing Li, Qian Wang, Cheng Zou

**Affiliations:** Institute for Advanced Materials and Technology, University of Science and Technology Beijing, Beijing 100083, China; m202411392@xs.ustb.edu.cn (J.G.); m202421618@xs.ustb.edu.cn (J.L.)

**Keywords:** transition metal carbides, rechargeable batteries, reversible capacity, electrode materials, carbide MXene

## Abstract

With the rapid development of electrical energy storage technologies, traditional battery systems are limited in practical applications by insufficient energy density and short cycle life. This review provides a comprehensive and critical summary of MXene or MXene-based composites as electrode materials for high-performance energy storage devices. By integrating the synthesis techniques of MXenes that have been studied, this paper systematically illustrates the physicochemical properties, synthesis strategies, and mechanisms of MXenes, and analyzes the bottlenecks in their large-scale preparation. Meanwhile, it collates the latest research achievements of MXenes in the field of metal–ion batteries in recent years, focusing on integrating their latest progress in lithium–ion, sodium–ion, lithium–sulfur, and multivalent ion (Zn^2+^, Mg^2+^, Al^3+^) batteries, and reveals their action mechanisms in different electrode material cases. Combining DFT analysis of the effects of surface functional groups on adsorption energy with experimental studies clarifies the structure–activity relationships of MXene-based composites. However, the development of energy storage electrode materials using MXenes and their hybrid compounds remains in its infancy. Future development directions for MXene-based batteries should focus on understanding and regulating surface chemistry, investigating specific energy storage mechanisms in electrodes, and exploring and developing electrode materials related to bimetallic MXenes.

## 1. Introduction

Over the past few decades, with rapid technological advances and the constant depletion of fossil fuels, there has been a growing demand for sustainable energy sources [1]. From various portable electronic devices to large-scale energy storage systems, rechargeable batteries have become the foundation of modern green life and progress [2]. However, current battery technology faces challenges such as low energy density, short cycle life, poor stability, and insufficient safety, which severely limits the application of batteries in high-power scenarios where they have to be charged and discharged quickly [3,4]. Therefore, it is urgent to further improve the performance of the battery, and traditional anode materials such as graphite are close to their theoretical capacity limits [5,6].

Metal–air batteries (zinc–air batteries) and metal–ion batteries (lithium, sodium, zinc plasma batteries) are the two major branches in the field of rechargeable batteries. Taking the zinc–air battery as an example, it stores energy through the electrochemical reaction between the zinc anode and oxygen in the air, and its theoretical energy density is much higher than that of a lithium–ion battery (5928 Wh/kg for lithium-air battery) [7]. However, its practical application is limited by the design of bifunctional catalysts (e.g., high cost of noble metal Pt/Ir and carbon carrier corrosion), CO_2_ sensitivity of the electrolyte, and cycling decay due to zinc dendrite growth [7]. In contrast, metal–ion eliminates the need for external oxygen and offers advantages in cycle life, power density, and process maturity.

Since its first report in 2011, MXene, an emerging two-dimensional material, has demonstrated remarkable potential in the field of electrochemical energy storage [8]. Characterized by a high specific surface area, excellent electrical conductivity, good mechanical properties, abundant surface functional groups, and a controllable layered structure, MXene offers higher theoretical capacity, faster ion transport efficiency, and better stability than traditional battery materials [9,10]. These advantages position MXene as a promising candidate for advancing next-generation energy storage technologies. However, there are still major challenges for its practical application. First, the process of large-scale preparation of MXene is immature, and most of the preparation methods are restricted to experiments. Second, MXene as an electrode material may produce accumulation between layers during charging and discharging, which may even lead to severe oxidative degradation at high current densities, affecting the performance of the battery [11,12]. Enhancing the interfacial compatibility of MXene with other active electrode materials to avoid side reactions is also one of the current keys to improve battery performance. To address these challenges, many strategies have been analyzed and proposed, including synthesizing and using a single extremely thin MXene lamella, extending the MXene lamella spacing to achieve tunability, constructing heterogeneous/composite electrode materials with MXene as the substrate and designing different electrode structures, and expanding the bridging between active materials and MXene [13,14]. By modifying and functionalizing MXene to improve its interfacial interaction with electrolytes, or by optimizing its surface functional groups to enable effective compositing with other high-capacity electrode materials, the cycling stability and charge transfer efficiency of MXene-based batteries can be significantly enhanced [15]. One of the most common applications of MXene is as a conductive substrate for composite electrodes, where its abundant surface functional groups enable the grafting or growth of other electrochemically active materials—such as transition metal oxides, sulfides, and other compounds—thereby constructing multidimensional conductive frameworks [15,16,17]. These materials are chemically synthesized by physical mixing, in situ growth, or solvothermal methods, or self-assembled into strong transition layers and covalently bonded heterogeneous structures by electrostatic interaction [18,19]. The special electronic structure and variable valence states of electrochemically active phases provide high-capacity storage sites, which effectively promote charge transfer and improve electrochemical performance [18,20].

**Figure 1:**
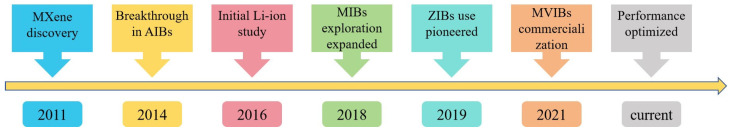
Timeline of representative MXene breakthroughs in MIBs in recent years.

In 2011, MXene was discovered and began to be noticed and used as a new material for metal–ion batteries; in 2014, a breakthrough was made in MXene-based aluminum–ion batteries (AIBs), which provided a direction for MXene to be explored in MVIBs; in 2016, preliminary lithium–ion research was launched, and MXene’s material properties were explored in depth; in 2018, MXene-based magnesium–ion batteries (MIBs) appeared and attracted attention; in 2019, the MXene-based zinc–ion battery (ZIB) was pioneered as an application of MVIB; in 2021, the multivalent¬–ion battery (MVIB) was commercialized, which could not be separated from the success of MXene’s many years of research accumulated in various ion battery systems. Currently, with the deepening of research, the performance of MXene combined with ion batteries has been optimized, and the application prospect is becoming more and more promising. These key points in chronological order clearly show the complete development of MXene and ion batteries from material discovery, and technological breakthroughs to application expansion and commercialization, as shown in Figure 1.

This review aims to systematically summarize the latest research advancements of MXene in the field of metal–ion batteries in recent years, detailing its structural-performance characteristics and synthesis methodologies. The synthesis approaches for MXene are categorized into three types based on fluorine usage: “fluorine-based etching”, “fluorine-free etching”, and “fluorine-free synthesis”. A comparative analysis is conducted to evaluate how different etching methods influence MXene’s surface functional groups and the number/thickness of its nanosheets. Additionally, the bottlenecks in large-scale MXene preparation are systematically analyzed, with a focus on deciphering the mechanisms of fluorine-free etching techniques to provide a theoretical foundation for green synthesis and functional modification. The review integrates the recent progress of MXene in lithium–ion, sodium–ion, lithium–sulfur, and multivalent ion (Zn^2+^, Mg^2+^, Al^3+^) batteries, revealing its multifunctional roles in different systems—such as anchoring polysulfides in lithium–sulfur batteries, regulating ion diffusion pathways, and inhibiting lithium dendrite growth. By combining density functional theory (DFT) analysis of surface group effects on adsorption energy with experimental results, the structure–activity relationships of MXene-based composites are clarified, highlighting the interplay between material structure and electrochemical performance. Finally, the review outlines future development directions for MXene-based batteries, proposing critical perspectives and suggestions to inspire research in electrochemical energy storage systems. These insights aim to guide advancements toward more efficient, sustainable, and high-performance energy storage technologies.

## 2. Properties of MXene

MXene materials have attracted considerable attention due to their exclusive properties, including high electrical conductivity, good mechanical properties, outstanding thermal stability, and hydrophilicity [21,22]. Moreover, they typically possess abundant surface functional groups (-OH, -O, -F, etc.), providing numerous active sites that endow them with remarkable surface reactivity and tunability. Coupled with their unique two-dimensional layered structure, these characteristics render MXene materials highly promising for wide applications in energy storage/conversion [23,24], sensors [25], catalysis [26], and electromagnetic shielding [27], etc.

### 2.1. Structure of MXene

**Figure 2:**
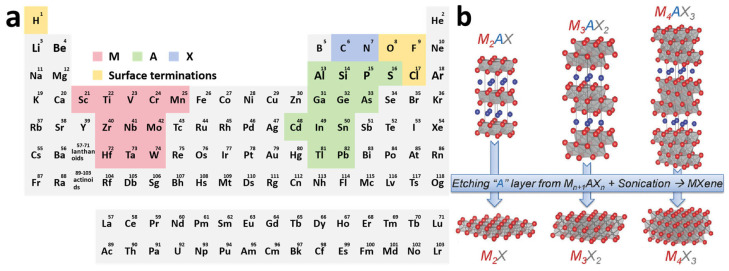
(**a**) Distribution of possible constituent elements of MXene in the periodic table. (**b**) Crystal structure of MXene obtained from MAX. Reproduced with permission [30]. Copyright 2013, Wiley-VCH.

MXenes are transition metal carbides, nitrides, and carbon–nitrides with an M_n+1_X_n_T_x_ formula, whose precursors were commonly designated as MAX phases [28]. As shown in Figure 2a, M represents early transition metal atoms, including any one of Sc, Ti, V, Cr, Mn, Zr, Nb, Mo, Hf, Ta, and W; A mainly consists of metallic and nonmetallic elements between the second and the fourth main groups, such as Al, Si, P, S, Ga, Ge, As, Cd, In, Sn, T, and Pb; X sites can be occupied by carbon, nitrogen, or both; *n* can vary from 1 to 4, and T_x_ (where x is variable) indicates surface terminations on the surface of the outer transition metal layers [29].

The M_n+1_A_n_X phase is a typical ternary layered ceramic, abbreviated as the MAX phase. MAX phases are classified into three distinct hexagonal crystal structures (space group P6_3_/mmc) according to the value of n, as 211, 312, and 413 [30,31]. It is well displayed in Figure 2b that the number of atomic layers between two layers of A atoms varies, and there is always one less layer of X atoms than M atoms during the periodic arrangement of M atoms under different structures. The MAX phase exhibits an alternating stacking sequence of A atom layers and M_(n+1)_X_n_ layers in space, where (n + 1) close-packed layers of M atoms form octahedral coordination polyhedra with X atoms filling the octahedral interstitial sites [30,32]. For example, Ti_2_AlC is the simplest MAX phase. It is formed by the alternate stacking of Ti-Al-Ti-C atomic layers in space. The C atoms are distributed between two adjacent layers of Ti atoms, composing a three-atomic-layer-arranged Ti-C-Ti structural microdomain. These microdomains are periodically intercalated by monolayer Al atomic sheets [33]. Among them, the M-X bond in the MAX phase has the mixed characteristics of ionic, covalent, and metallic bonds, while the M-A bond is purely metallic in nature. Since the bond energy of the M-A bond is relatively weaker than that of the M-X bond, the M-X atomic layers can be retained by chemically etching the M-A bond, thereby obtaining a two-dimensional layered structure similar to graphene. However, unlike graphene, the M-A bond still belongs to a chemical bond, and its bond energy is much greater than the van der Waals force between the two-dimensional graphene layers, so it is difficult to separate it by mechanical methods [34]. About the predominant approach for MXene synthesis, the A layers with relatively weak bond energy in the MAX phase can be selectively etched by chemical means and replaced by active functional groups, resulting in three-layer or multi-layer M-X-M nanosheets with various functional groups on the surface, called M_n+1_XT_x_.

### 2.2. Conductivity

In 2004, Geim and Novoselov first isolated single-layer graphene, marking the advent of the two-dimensional materials era [35]. Two-dimensional materials have highly efficient heat and charge transfer in the in-plane direction. MXene is not only structurally similar to two-dimensional graphene, but its metal layers also possess excellent conductive properties [35]. MXene surface functional groups have a strong influence on the electrical conductivity. When electrons are transferred from M-metal to the surface of MXene, the Fermi energy level of M_n+1_X_n_T_x_ without surface functional groups will move downward, and the electrical conductivity will be reduced, and all MXenes without surface groups theoretically exhibit metallic properties. Therefore, the conductivity of MXenes can be adjusted by adjusting the type and number of surface functional groups [36,37]. R et al. [38] employed hydrogen plasma treatment to make the -OH groups on the surface of MXene react and neutralize with H atoms, obtaining the surface-exposed Ti_3_C_2_ MXene without terminal groups. It was found that the resistivity of MXene decreased from 5.64 μ Ω m to 4.62 μ Ω m. Subsequently, O_2_ plasma treatment was carried out to form new functional groups (=O) on the surface, and at this time, the resistivity increased to 5.71 μ Ω m. During the etching process, the M-A bonds will break, and the outermost electrons of the surface metal atoms are in an approximately unmatched transition state. At this moment, the surfaces of some MXenes will be terminated and transformed into semiconductors [38,39]. Therefore, the high conductivity of MXene materials mainly depends on the density of states near the Fermi level of the M-layer atoms. When the surface functional groups of MXenes are the same, their electrical conductivity mainly depends on the type of M atoms in MXene.

Moreover, in addition to the reactive groups with M atoms, X atoms also have a large effect on the electrical conductivity of MXene materials. Zhang et al. [40] calculated the DOS of Ti_n+1_C_n_T_2_ and Ti_n+1_N_n_T_2_ at the Fermi energy level (EF) (T = -F, =O, -OH; n = 1,2,3). The results show that the Fermi energy levels of the nitride MXenes are higher than those of the carbide MXenes regardless of the termination groups and that the nitride MXenes have higher electrical conductivity which can be attributed to the fact that N possesses more electrons than C [40,41]. This is due to the fact that the M and X atoms of MXene are covalently bonded, and the electrons within the covalent bond form an energy band structure, with the discrete electrons moving through the valence band to produce electrical conductivity. The N atom has more electrons than the C atom, which gives the nitride MXene a higher electrical conductivity [42].

### 2.3. Hydrophilicity

The principal factors defining the hydrophilicity of MXenes are the surface terminal groups’ hydrophilic capability. Therefore, the rational selection of the etching process is critical, as the chemical etching of MAX phases generates abundant hydrophilic functional groups (-OH, -O, and -F terminations) that can form a large number of hydrogen bonds with water molecules [43]. In aqueous ion batteries, these hydrogen bonds interact with electrolyte solvent molecules, significantly enhancing the electrode material’s wettability toward the electrolyte, and lead to the reduction of interfacial contact resistance, which consequently accelerates the transport of hydrated ions [44,45]. Notably, in multivalent ion batteries (MIBs), the electrostatic screening effect induced by polar functional groups can effectively suppress the Coulombic repulsion during the intercalation of high-valence ions (HVIs). In addition, hydrogen bonding between these hydrophilic functional groups reinforces interlayer adhesion to alleviate volume expansion and structural degradation during cycling, and enables hydration-mediated interlayer spacing expansion, which creates diffusion channels for large-radius ions (K^+^, Zn^2+^) and thereby enhances rate performance [44,45,46]. It is understood from the mentioned findings that the pronounced hydrophilicity of MXenes ensures both stable colloidal dispersion and rapid ion transport kinetics in aqueous electrolytes. For non-aqueous systems, however, precisely balanced amphiphilicity is required to prevent detrimental water absorption that would trigger particle aggregation or parasitic side reactions. Most nanomaterials exhibit intrinsic hydrophobicity, leading to mutual aggregation driven by van der Waals forces, which severely restricts their further applications. Consequently, MXenes emerge as a promising candidate for diverse nanocomposite architectures.

### 2.4. Mechanical Properties

The MXene lamellae themselves exhibit excellent mechanical properties due to their unique structure [47]. This is because after the A layer atoms are chemically etched away, the electrons are more concentrated between the M-phase and the X-phase. As mentioned above, the M-X bond is a chemical bond that is a mixture of multiple bonds and tends to be metallic, with a high binding strength. Its tensile stiffness ranges from 81.71 to 561.4 N/m, surpassing traditional two-dimensional materials including graphene [48]. It is worth noting that after etching, MXene can be transformed from multi-layer accordion-like MXene into single-layer or few-layer nanosheets by inserting organic molecules or other metal ions between the layers [49]. When the constituent elements are the same, the number of layers of different MXene lamellae will also affect their mechanical properties. However, due to the extremely small size of MXene nanosheets, the problem of stacking is highly likely to occur, leading to a decline in mechanical and electrochemical properties. Adding conductive spacers between MXene lamellae to construct a special layered structure is one of the effective ways to solve the above problems and effectively improve the mechanical properties of MXene-based composites. Jiao et al. successfully constructed a continuous honeycomb-like conductive network structure, a waterborne polyurethane/natural rubber/Ti_3_C_2_T_x_ (WPU/NR/MXene) composite film with flexibility and certain self-healing properties, by using electrostatic repulsion interaction and vacuum-assisted filtration [50]. Although the content of MXene is only 5.48 vol% compared with the WPU/NR film without MXene, the WPU/NR/MXene composite film has an increase of 10.3 times and 8.8 times in tensile strength and toughness, respectively. Similarly, Luo et al. employed a double-crosslinking method. They crosslinked and modified MXene with dopamine, and then introduced Ca^2+^ in situ into the obtained layered composite film to further form ionic bond crosslinking between CNF (Cellulose Nanofibrils) and MXene [51]. The results showed that this double-crosslinking composite film strategy enhanced both the toughness and strength of the film, increasing them from 72.66 MPa and 3.85 MJ/m^3^ to 142.2 MPa and 9.48 MJ/m^3^, respectively, significantly improving the mechanical stability.

## 3. Preparation Methods

The preparation of two-dimensional MXenes is mainly obtained by treating their precursor MAX phase. As can be seen from the above, in the MAX phase, the metallic M-A and M-X bonding energies are much larger than the van der Waals forces, which makes it difficult to fabricate MXenes by conventional mechanical stripping techniques; however, the metallic M-A bonding is weaker than that of the M-X bonding, which has a mixed bonding, and therefore the “A” layer can be selectively removed to form the M_n+1_X_n_T_x_ phase [30]. In 2011, Professor Gogotsi’s team at Drexel University successfully obtained an accordion-shaped Ti_3_C_2_T_x_ MXene through the use of HF etching of the aluminum atomic layer in Ti_3_AlC_2_ MAX, thereby paving the way for significant advancements in MXene materials research and applications [8]. Later, various etching methods have been used to prepare MXene with different structures, and these different etching methods can be categorized into two main groups according to whether they contain fluorine or not. In the early stage, the etchant for preparing MXene mainly involves fluorine-containing compounds, such as HF, LiF + HCl, and fluorine-containing molten salts [52]. Due to the properties of these compounds, three key surface termination groups of MXenes were identified, namely -F, -OH, and -O groups. Fluorine-containing etching methods appeared the earliest, with high yields suitable for large-scale production and various uses, including and not limited to HF etching, in situ generation of HF etching, fluorine-containing molten salt etching, and so on; as opposed to the earliest production of non-fluorinated MXenes using electrochemical etching as early as in 2017, and with this there has been the emergence of other fluorine-containing etching such as alkali etching, non-fluorine-containing molten salt etching, and recently emerging alternative fluorine-containing etching such as Lewis acid molten salt etching, and other emerging methods of substituting fluorine-containing etching [53,54,55]. In addition to the top-down etching methods mentioned above, CVD (chemical vapor deposition) for assembling MXene nanomaterials tailored to specific properties from atomic or molecular sizes is considered a special bottom-up synthesis method [56]. In this paper, MXene synthesis methods are categorized in detail according to the degree of fluorine usage. And the effects of different etching methods on the functional groups on the surface of MXene and the number/thickness of laminae were compared, as shown in Table 1.

### 3.1. Etching with HF and F Salts

**Figure 3:**
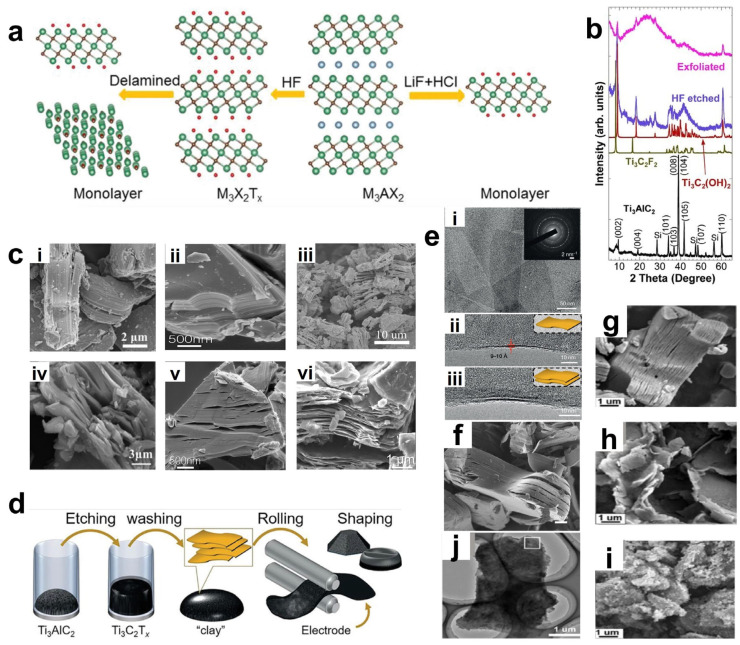
(**a**) Schematic of the principle of using HF etching or in situ generation of HF etching. Green, light blue, brown, and red balls represent M, A, X, and T atoms, respectively. XRD images of Ti_3_AlC_2_ before HF treatment (simulated as XRD of Ti_3_C_2_F_2_ and Ti_3_C_2_(OH)_2_) and after HF treatment and measured by ultrasonic stripping. Reproduced with permission [58]. Copyright 2022, The Royal Society of Chemistry. (**b**) XRD images of Ti_3_AlC_2_ before HF treatment (simulated as XRD of Ti_3_C_2_F_2_ and Ti_3_C_2_(OH)_2_ and after HF treatment and measured by ultrasonic stripping. Reproduced with permission [57]. Copyright 2011, Wiley-VCH. (**c**) SEM images of various MXene and MAX: (**i**) Ti_2_AlC(MAX). Reproduced with permission [60]. Copyright 2023, Springer Nature. (**ii**) Mo_2_TiAlC_2_(MAX). Reproduced with permission [65]. Copyright 2015, American Chemical Society. (**iii**) Ti_3_C_2_T_x_. Reproduced with permission [59]. Copyright 2015, American Chemical Society. (**iv**) Ti_2_CT_x_. Reproduced with permission [68]. Copyright 2013, American Chemical Society. (**v**) Mo_2_TiC_2_T_x_. Reproduced with permission [65]. Copyright 2015, American Chemical Society. (**vi**) V_2_CT_x_. Reproduced with permission [61]. Copyright 2013, American Chemical Society. (**d**) Schematic of the process: etching of the MAX phase in a solution of acid and fluoride salts and washing with water to adjust the pH to neutrality, the resulting precipitates have a clayey character. (**e**) (**i**) TEM image of a few thin sheets of Ti_3_C_2_T_x_ with transverse dimensions up to a few hundred nanometers. Very few defective regions are present. The inset shows the electron diffraction pattern of the entire selected region (**ii**,**iii**) TEM images of single and bilayer flakes in the longitudinal direction, respectively. (**f**) Accordion-like multilayered MXene particles. (**d**–**f**) Reproduced with permission. Reproduced with permission [69]. Copyright 2014, Springer Nature. (**g**–**i**) SEM images of (**g**) 8 h, (**h**) 16 h, and (**i**) 32 h etched Ti_3_AlC_2_ MAX phase and corresponding h-Ti_3_C_2_ MXene. (**j**) TEM micrograph of h-Ti_3_C_2_ flakes after 16 h etching. (**g**–**j**) Reproduced with permission [70]. Copyright 2022, Elsevier.

Naguib et al. first adopted HF to etch Ti_3_AlC2 [57]. The mechanism is shown in Figure 3a. Relying on the relatively strong reactivity between F ions and Al ions, the M-Al bonds are broken to form AlF_3_, thereby selectively etching away the aluminum layer. This process is accompanied by the generation of hydrogen gas at the same time, and finally the accordion-like MXene material is obtained [58]. The layers are mainly connected by van der Waals forces and hydrogen bonds of surface active groups. The reaction equation is shown as follows:

Ti_3_AlC_2_ + 3HF = AlF_3_ + 1.5H_2_ +Ti_3_C_2_ (1)

Ti_3_C_2_ + 2HF = Ti_3_C_2_(F)_2_ + H_2_ (2)

Ti_3_C_2_ + 2H_2_O = Ti_3_C_2_(OH)_2_+ H_2_ (3)

Ti_3_C_2_ + 2H_2_O = Ti_3_C_2_(O)_2_+ 2H_2_ (4)

First, the Al layer is selectively removed from the Ti_3_AlC_2_ MAX phase containing Al to generate an unsaturated structure of Ti_3_C_2_. After the Al atoms are selectively etched away by F ions, the Ti atoms with unsaturated charges on the surface of Ti_3_C_2_ are highly prone to bond with atoms such as -OH, -F, and -O, endowing its surface with abundant -OH and -F groups, thus generating a rich group of surface functional groups. With the in-depth progress of research, people have successfully etched a variety of MAX phases based on HF, resulting in many new MXene materials, such as Ti_3_C_2_T_x_ [59], Ti_2_CT_x_ [60], V_2_CT_x_ [61], Nb_2_CT_x_ [62,63], Zr_3_Al_3_C_5_ [64], Ta_4_C_3_T_x_ [64], and Mo_2_C_2_T_x_ [65,66]. As shown in Figure 3c, the morphologies of several MAX phases (Ti_2_AlC, Mo_2_TiAlC_2_) and several MXene phases (Ti_3_C_2_T_x_, Ti_2_CT_x_, Mo_2_TiC_2_T_x_, V_2_CT_x_) are presented. It can be observed that they all exhibit similar accordion-like morphological characteristics, indicating that the products after etching with hydrofluoric acid possess a certain universality. Figure 3b shows the XRD patterns of the sample Ti_3_AlC_2_ before and after etching with hydrofluoric acid [57]. The disappearance of the characteristic peak at 39° is due to the fact that HF selectively etches away the Al layer in Ti_3_AlC_2_, causing the remaining Ti_3_C_2_ structure to be reorganized or deformed, and thus the original characteristics of the crystal structure disappear. Meanwhile, the diffraction peak of the (002) crystal plane shifts to a lower angle. According to the Bragg equation (nλ = 2d sinθ), the decrease in the diffraction angle means an increase in the interlayer spacing d. This increase may be attributed to the fact that the removal of the Al layer reduces the charge-balancing force between the layers, thereby weakening the force between the Ti_3_C_2_ layers and leading to an expansion of the interlayer spacing in the crystal structure. All these indications prove the generation of the new two-dimensional material MXene.

However, it is often difficult to obtain the desired single-layer MXene by this method. The greater the bond energy between M and A, the more and thicker the MAX phase lamellae are, the stricter the etching conditions with HF will be, and the lower the yield will be [67]. Although the direct use of HF etching has the advantages of simple process and high yield, the use of highly hazardous, highly polluting, and highly corrosive HF in the etching process is increasingly inconsistent with the concept of green development. Therefore, greener and safer preparation methods are needed for substitution.

Based on the etching mechanism, researchers first came up with the idea of replacing the direct use of HF acid with the method of generating HF in situ during the etching process. Fluoride salts (such as LiF, NaF, KF, NH_4_F, etc.) are mixed with hydrochloric acid or sulfuric acid, etc. Similarly, the F ions are utilized to interact with the A atoms in the MAX phase to generate fluorides. It is worth noting that the cations in the fluoride salts will act as mild intercalating agents to insert between the lamellae to reduce the van der Waals forces, thereby obtaining few-layer or even single-layer MXene nanosheets [69]. However, since these cations act as mild intercalating agents, single-layer nanosheets can be efficiently obtained only when the van der Waals forces between the nanosheet layers are relatively small, that is, when there are only two or a few metal atom transition layers. Ghidiu et al. first used a mixture of HCl and LiF to prepare MXene [69]. The flowchart is shown in Figure 3d. Ti_3_AlC_2_ powder was etched at 40 °C, and then exfoliated through dimethyl sulfoxide intercalation and ultrasonic vibration, resulting in single-layer to multi-layer Ti_3_C_2_T_x_ MXene flakes (Figure 3e shows the TEM images of single-layer and double-layer nanosheets). Observed under a transmission electron microscope (TEM), it was found that the HCl/LiF mixture is similar to HF, and a similar accordion structure was also produced during the etching process. Moreover, the lateral size of the Ti_3_C_2_T_x_ flakes reaches from hundreds of nanometers to several micrometers, and the thickness is between 1 nm and dozens of nanometers (Figure 3f). Notably, the etching duration exerts profound influence on the resultant microstructure. When employing NaBF_4_/HCl as the etchant, multi-layer 2D flakes were obtained after 8 h (Figure 3g), while ultrathin nanosheets formed at 16 h (Figure 3h). Prolonged etching to 32 h yielded AlF_3_-coated agglomerates (Figure 3i), indicating structural degradation and undesirable byproduct formation. TEM characterization revealed transparent Ti_3_C_2_ nanosheets with optimal structural integrity after 16 h etching (Figure 3j), demonstrating this duration represents the processing optimum. Compared with HF etching, this method is milder, and the obtained MXene flakes have larger sizes and fewer defects, significantly optimizing the electrochemical properties and application potential of the material.

**Figure 4:**
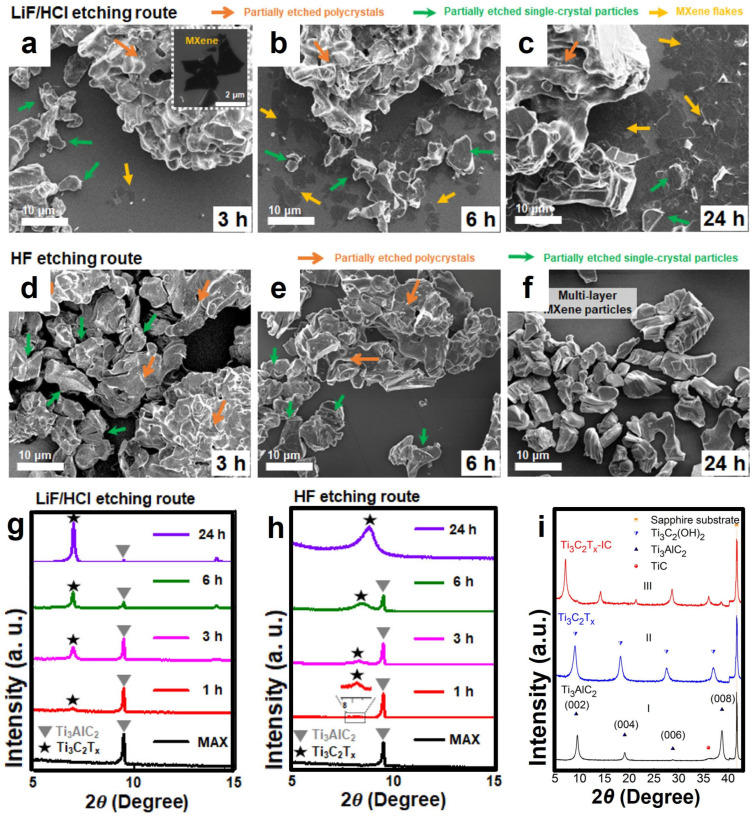
(**a**–**c**) The SEM images of the samples etched by LiF/HCl for different durations, corresponding to the samples etched by HF for (**d**) 3 h, (**e**) 6 h, and (**f**) 24 h, respectively. (**g**) The XRD patterns of the samples etched by LiF/HCl and (**h**) those etched by HF. Reproduced with permission [71]. Copyright 2021, American Chemical Society. (**i**) (I) The XRD pattern of the Ti_3_AlC_2_ film. (II) Ti_3_C_2_T_x_ after being etched in 50% HF for 2 h and 40 min. (III) Ti_3_C_2_T_x_ -IC after being etched in 1 M NH_4_HF_2_ for 11 h. Reproduced with permission [72,73]. Copyright 2014, American Chemical Society.

To further explore the mechanism of the etching process of Ti_3_AlC and the intercalation effect of cations, Kim et al. summarized different fluorine-containing etching methods such as HF, NH_4_HF_2_, and LiF/HCl, and proposed that the etching proceeds step by step, that is, the etching of Al atoms in the inner layer only occurs when the adjacent outer layer is exfoliated [71]. As shown in Figure 4a–c, the SEM images of the HF etching process reveal that with continuous etching, the larger polycrystalline Ti_3_AlC_2_ particles gradually decompose into smaller single-crystal particles. After etching for 24 h, only single-crystal particles remain. By comparing the SEM images of the LiF/HCl etching process (Figure 4d–f), a phenomenon similar to that of HF etching can be observed, where the polycrystalline aggregates break down into finer crystals. However, compared with Ti_3_C_2_T_x_ prepared by HF etching, the interlayer distance of multi-layer Ti_3_C_2_T_x_ is significantly increased. Analyzing the XRD patterns of the two methods (Figure 4g,h), the complete disappearance of the (002) peak of the MAX phase etched by HF indicates that the exfoliation of the Ti_3_C_2_T_x_ layer has not been triggered. Similarly, NH_4_HF_2_ etching has an effect similar to that of HF, but NH_4_HF_2_ provides the non-fluorine cation NH_4_^+^ as an intercalating agent to enter the MXene layer and forms a water-soluble byproduct (NH_4_)_3_AlF_6_, and the interlayer distance of its multi-layer MXene is larger than that of HF. The same conclusion can be drawn from the analysis of the XRD pattern of Ti_3_C2Tx [72]. Figure 4i (II) shows the shift of the 000 peak to a lower angle, corresponding to the increase of the C lattice parameter from 18.6 Å of Ti_3_AlC_2_ to 19.8 Å of Ti_3_C_2_T_x_. In the XRD pattern of Ti_3_C_2_T_x_-IC (III, where IC represents the inserted species, namely NH_3_ and NH_4_^+^), the C lattice further increases to 24.7 Å. This indicates that the C lattice constant of Ti_3_C_2_T_x_ etched by NH_4_HF is 25% larger than that of Ti_3_C_2_T_x_ etched by HF.

It is worth noting that HF etching will attack the grain boundaries, causing the polycrystals to break into single crystals, with higher etching efficiency. However, it is difficult to obtain single-layer MXene nanosheets due to the lack of intercalating agents. When LiF/HCl is used for etching, initially only the surface reacts and most of the internal regions remain unetched. Moreover, during the delamination process of Li+ and water molecules embedding into the MXene flakes, incomplete exfoliation or impurity formation may occur, leaving incompletely etched MXene particles. Therefore, although the method of generating HF in situ significantly reduces the hazards caused by toxicity and high corrosiveness, it is only suitable for most MAX phases containing Al as the A atom layer, and the yield of fluoride ions is lower compared with the direct use of HF, and the etching range is limited. To completely convert the MAX phase into MXene, the etching conditions need to be adjusted rigorously. Research shows that the etching conditions are related to the atomic number of the M atom. Usually, the larger the atomic number, the higher the corresponding increase in the M-A bond energy, and the more stringent the required F ion concentration and etching time will be.

### 3.2. Alkali Etching

In order to avoid the harm caused by fluorine and improve the etching precision and the conductivity of MXene, researchers have further investigated the fluorine-free etching method known as the alkali etching method. Under a high temperature and high-concentration alkaline environment, the A phase containing amphoteric metal atoms, such as Al atoms, will react with OH- to generate the corresponding hydroxide, that is, Al(OH)^4−^ ions, which will dissolve in the alkaline solution, thereby being removed from the MAX phase, and -OH functional groups will be formed on the surface of the transition metal layer by combination [71,72]. This process is endothermic. According to the principles of thermodynamics, a relatively high reaction temperature is one of the important factors affecting etching. Li et al. first achieved the synthesis of high-purity MXene by using the alkali etching method and explored the two main factors affecting hot alkali etching—temperature and alkali concentration [72,74]. They used a hot NaOH solution with a concentration of 27.5 mol/L at 270 °C to etch Ti_3_AlC_2_ and successfully prepared multi-layer Ti_3_C_2_T_X_ without fluorine on the surface. The specific chemical reaction equations are as follows:

Ti_3_AlC_2_ + OH^−^ + 5H_2_O → Ti_3_C_2_(OH)_2_ + Al(OH)^4−^ + 2.5H_2_ (5)

Ti_3_AlC_2_ + OH^−^ + 5H_2_O → T_i3_C_2_O_2_ + Al(OH)^4−^ + 2.5H_2_ (6)

**Figure 5:**
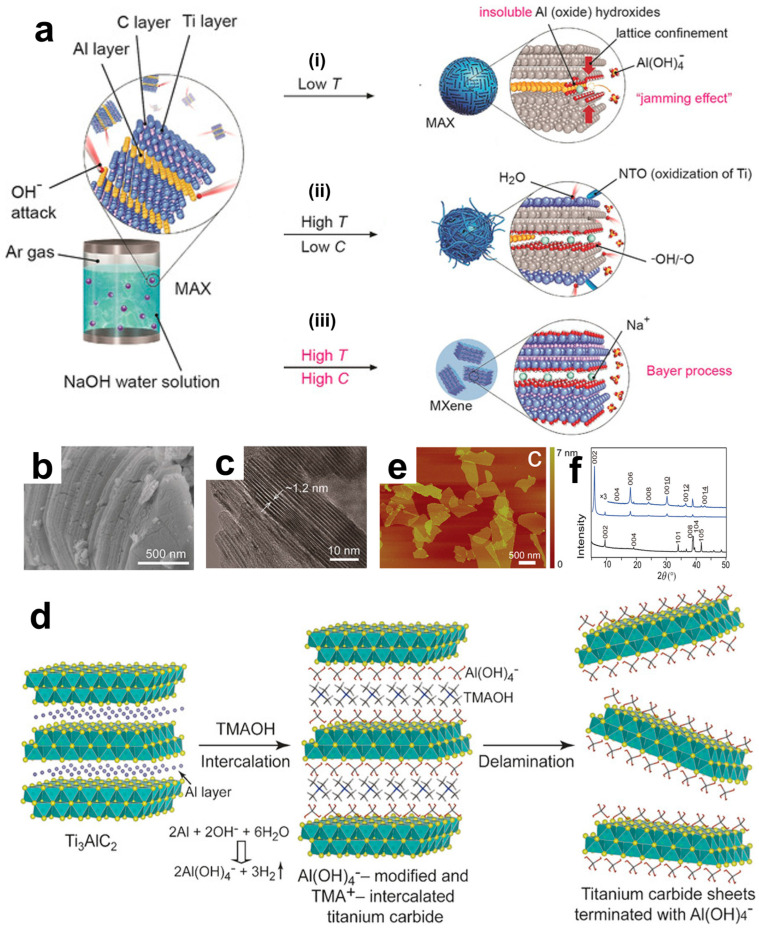
(**a**) The etching process of Ti_3_AlC_2_ with NaOH aqueous solution under three different conditions. (i) Low temperature: at low temperature, insoluble Al oxides/hydroxides are formed. (ii) High temperature, low carbon: under high temperature and low carbon environment, H_2_O is involved in the reaction, Ti is oxidized to form NTO, OH- and -O are involved in the reaction process. (iii) High-temperature, high-carbon: MXene is finally formed. Through (**b**) SEM and (**c**) TEM for the microscopic structure observation of Ti_3_C_2_T_x_ flakes, where the bright spots in the figures indicate the positions of Ti. (**a**–**c**) Reproduced with permission [74]. Copyright 2018, Wiley-VCH. (**d**) The intercalation and delamination process of organic bases, demonstrating the representative bright-field of extremely thin nanosheets. (**e**) The AFM image after the intercalation and separation of Al(OH)^4−^. (**f**) The XRD patterns of the samples before and after the reaction in aqueous TMAOH, indicating that the basal plane spacing expands from 0.92 to 1.50 nm (**d**–**f**). Reproduced with permission [75]. Copyright 2016, Wiley-VCH.

It was found that as the temperature decreases, the production rate of soluble Al(OH)^4−^ will be greatly reduced, thereby significantly reducing the yield of MXene. It is even difficult to produce MXene at 220 °C. In addition, according to chemical kinetics, the concentration of hydroxide ions is another important factor affecting etching. Theoretically, the lower the concentration of hydroxide ions, the correspondingly lower the etching rate and yield. This is also the case in practice. The lower the concentration of NaOH and the higher the water content, the easier it is to cause the oxidation of Ti and generate a series of titanium oxides. Eventually, Li et al. found that by hydrothermal treatment with 27.5 M NaOH at 270 °C, multi-layer Ti_3_C_2_T_x_ with a purity close to 92% could be obtained. As shown in Figure 5a (i), at low temperatures, aluminum hydroxide is difficult to transform into soluble ions. (ii) At high temperatures but with low OH^−^ concentrations, the higher water content leads to the oxidation of MXene and the generation of a large amount of oxide impurities. (iii) According to the Bayer process, the high temperature and high NaOH concentration of 27.5 M NaOH at 270 °C can most efficiently convert and dissolve aluminum or its oxides. Observing its surface morphology, as shown in Figure 5b,c, similar to Ti_3_C_2_T_x_ treated with low-concentration HF, the prepared Ti_3_C_2_T_x_ has a compact accordion-like structure. Besides using strong alkalis, milder and safer organic alkalis as etchants can also obtain relatively pure products. And due to the significant intercalation effect of organic cations, the thickness of the obtained nanosheets can be reduced. Based on the mechanism of hydroxide ions etching aluminum atoms, Xuan et al. used tetramethylammonium hydroxide (TMAOH) as an etchant, and the process is schematically shown in Figure 5d [75]. TMAOH reacts with the Al atom layer between the layers, causing the hydrolysis and breakage of the Ti-Al bond. The generated Al(OH)^4−^ will modify the etched surface, and at the same time, the bulky TMA will be inserted between the layers as an intercalating agent. Looking at the AFM image in Figure 5e, ultimately, uniformly distributed and highly delaminated ultrathin nanosheets are obtained. Through X-ray diffraction analysis (Figure 5f), after the reaction in the TMAOH solution, new diffraction peaks are observed, indicating that the interlayer spacing has increased to 1.50 nm, successfully realizing the intercalation of TMA ions.

Alkali etching also has considerable disadvantages. When the concentration of the alkali solution and the reaction temperature are relatively low, the corrosiveness to MAX is relatively weak, which easily causes the oxidation of the M and X skeletons, greatly reducing the integrity of the MXene structure, and is accompanied by the problem of insufficient reaction kinetics, only being able to etch its surface [64]. If the reaction temperature is too high, it may also bring certain safety hazards.

### 3.3. Electrochemical Etching Method

The electrochemical etching method is one of the fluorine-free etching methods. The principle lies in utilizing the conductivity of the MAX phase to apply a specific voltage to both ends of the MAX electrode in the electrolyte, thereby selectively etching away the A atom layer [76].

**Figure 6:**
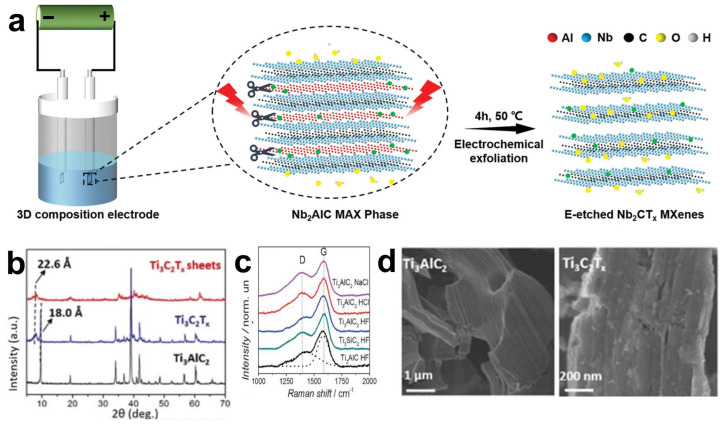
(**a**) A schematic illustration of the exfoliation and delamination process of the Nb_2_AlC MAX phase via electrochemical etching. The red lightning symbol indicates electrical drive. Reproduced with permission [78]. Copyright 2020, Wiley-VCH. (**b**) The X-ray diffraction patterns of Ti_3_AlC_2_, Ti_3_C_2_T_x,_ and Ti_3_C_2_T_x_ thin films. Reproduced with permission [77]. Copyright 2018, Wiley-VCH. (**c**) The Raman spectra of amorphous carbon CDC synthesized by etching the MAX phase (namely Ti_3_SiC_2_, Ti_3_AlC_2_ and Ti_2_AlC) in HCl, NaCl or HF electrolytes. The letters D and G represent Raman spectral peaks, the G peak corresponds to the vibration in the sp^2^ hybridized carbon plane, and the D peak is the defect-induced peak. Reproduced with permission [80]. Copyright 2014, Wiley-VCH. (**d**) The representative SEM images of Ti_3_AlC_2_ and Ti_3_C_2_T_x_. Reproduced with permission [77]. Copyright 2018, Wiley-VCH.

In 2018, Yang et al. first reported a fluoride-free electrochemical method. By using a binary aqueous electrolyte (0.1 M NH_4_Cl and 0.2 M TMAOH), a voltage was applied to the surface of the Ti_3_AlC_2_ electrode to selectively break the Ti-Al bonds, and the aluminum layer was successfully etched away [77]. Song and their team also adopted this fluoride-free electrochemical etching method and successfully synthesized 2D fluoride-free Nb_2_CT_x_ MXene nanosheets in a standard three-electrode configuration [78]. The specific schematic diagram of the principle is shown in Figure 6a. It is worth noting that it is usually considered that the chloride ions in the electrolyte are the key to this method. When a voltage is applied, the Cl ions at the positive electrode can easily react with the A atom layer to selectively etch away the A layer and attach -OH end groups on the surface of the transition metal layer, forming M_n+1_A_n_(OH)_X_. However, there must be necessary catalysts in the electrolyte, so the surface of the produced MXene does not only have the -OH group, but also is related to the types of groups carried by the catalysts, such as -Cl, -F, and -O, etc. Figure 6c shows the Raman spectra of three MAX phases, namely Ti_3_AlC_2_, Ti_3_SiC_2_, and Ti_2_AlC, which are successfully converted into amorphous carbon CDCs in different electrolytes. When Ti_3_AlC_2_ is etched using dilute electrolytes such as NaCl, HCl, and HF, both titanium and aluminum atoms may be removed, thus generating amorphous carbon. While using a medium-concentration HCl solution will lead to the selective aluminum extraction on the surface of the bulk Ti_3_AlC_2_ and form a thin protective carbon layer on the surface, preventing the further progress of etching [79]. Meanwhile, the binary electrolyte TMAOH adopted by Yang et al. [77] was added as a certain amount of intercalating agent, which enlarged the distance between the lamellae, promoting a larger specific surface area inside the MAX phase to come into contact with the electrolyte, thereby reducing the differences caused by the inconsistent etching speeds between the surface and the interior. The XRD pattern in Figure 6b reveals the structural changes during the etching and delamination processes. The (002) peak of Ti_3_AlC_2_ shifts to a lower angle, corresponding to the increase of the C lattice parameter from 18.0 to 22.6 Å, indicating that TMAOH can be easily embedded into the interlayer of the MAX phase, thus increasing the efficiency of the electrolyte in etching the Al layer. When observing the SEM images (Figure 6d), it is found that after electrochemical etching, the multi-layer Ti_3_C_2_T_x_ powder is still tightly stacked, similar to the compact layered bulk Ti_3_AlC_2_, rather than the accordion-like structure produced by traditional hydrofluoric acid etching. This is because when a voltage is applied to both ends of the MAX precursor, and the precursor has a large volume and dense layers, the MAX phase on the surface layer close to the electrolyte is often preferentially converted into the MXene phase, while the middle part remains the MAX phase, which significantly reduces the yield.

In addition, during the etching process, besides selecting an appropriate electrolyte, it is also necessary to choose a suitable voltage so as to avoid the possible Ti etching phenomenon under high polarization and the formation of carbide-derived carbon (CDC). Liu and their team treated the prepared TiAlC MAX phase [81]. Through the in situ molten salt electrochemical etching method under low voltage conditions (1.3 V versus ref.), Al atoms were selectively removed from the MAX phase, and Ti_2_CT_x_ MXene was successfully prepared. During this process, these Al atoms were oxidized to Al^3+^ and deintercalated from the Ti_2_AlC phase. Meanwhile, the Al^3+^ ions in the LiCl/KCl molten salt formed a volatile AlCl_3_ phase. The free Cl^−^ in the molten salt will fill the vacant positions of Ti_2_C to form surface terminal groups -Cl. In addition, the free Li^+^/K^+^ ions in the molten salt will move towards the negative electrode and be reduced at the negative electrode to form Li/K metal deposits.

### 3.4. Molten Salt Etching

**Figure 7:**
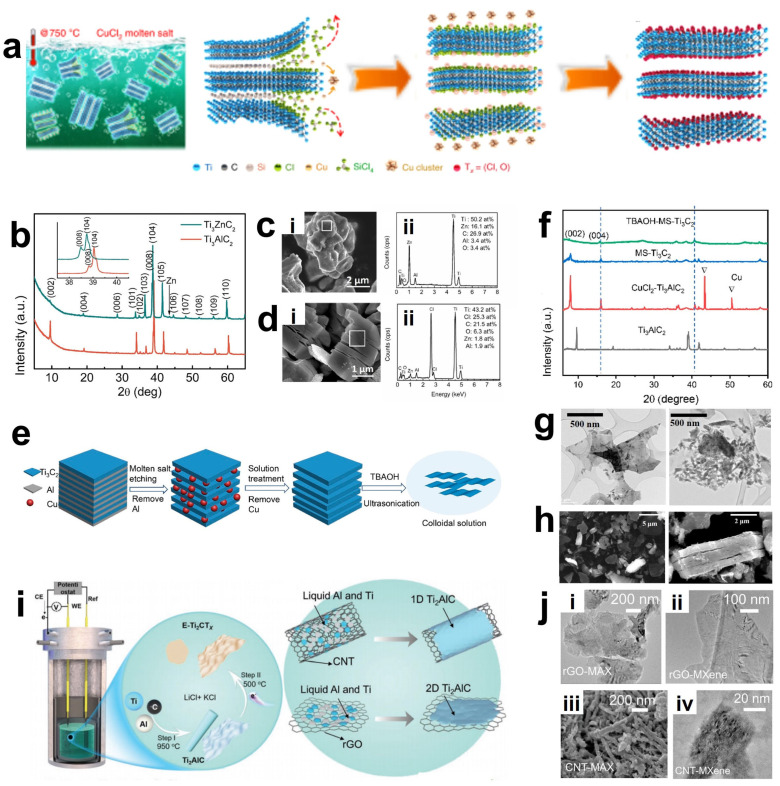
(**a**) Schematic diagram of Ti_3_C_2_T_x_ MXene preparation. Reproduced with permission [86]. Copyright 2020, Springer Nature. (**b**) XRD patterns of Ti_3_AlC_2_ and Ti_3_ZnC_2_. (**c**) SEM image (**i**) of Ti3ZnC2 and the corresponding energy-dispersive spectroscopy (EDS) spectrum (**ii**). (**d**) SEM image (**i**) of Ti3C2Cl2 and the corresponding EDS spectrum (**ii**). (**b**–**d**) Reproduced with permission [85]. Copyright 2019, American Chemical Society. (**e**) Molten salt synthesis method and exfoliation process. (**f**) X-ray diffraction (XRD) patterns of MAX phase precursors (black), MXene after removal from the molten salt bath (red), MXene after washing in APS to remove residual Cu (blue), and MXene after TBAOH stripping treatment (green). The triangular symbols indicate the characteristic diffraction positions where Cu^2+^ is reduced to Cu monomers remaining in the sample. (**g**,**h**) SEM maps of MS-Ti_3_C_2_T_x_ MXene treated with DMSO followed by sonication, and MS-Ti_3_C_2_T_x_ MXene treated with TBAOH for 24 h and 72 h TEM images of MS-Ti_3_C_2_T_x_. (**e**–**h**) Reproduced with permission [87]. Copyright 2021, American Chemical Society. (**i**) Schematic diagram of a molten salt cell with (Ti, Al, C) pellets as working electrode, glassy carbon as reference electrode, and graphite crucible as counter electrode and a schematic diagram of the process for the preparation of Ti_2_AlC by using carbon nanotubes and reduced graphene oxide as the carbon source. (**j**) Ti_2_AlC MAX (**i**,**iii**) obtained by using graphene oxide and carbon nanotubes as the carbon source and Ti_2_CCl_2_ MXene (**ii**,**iv**) TEM images of the Ti_2_CCl_2_ MXene. (**i**,**j**) Reproduced with permission [81]. Copyright 2022, Wiley-VCH.

Most methods for obtaining MXene require the etching of Al atoms in aqueous systems. However, for MAX precursors without an Al layer or MAX phases containing nitrides, the etching process often needs to overcome a higher energy barrier, which significantly limits the types of MXene [82]. Therefore, researchers have explored placing the MAX phase precursors in molten salts with certain compositions. At this time, the A atom layer will be replaced by the atoms in the molten salts without changing the structural frameworks of M and X. Accordingly, it is possible to etch some MAX phases that are difficult to etch by traditional methods, such as those with the A atom layer composed of Si and some nitride MAX phases [83,84]. Li et al. [85] reported this preparation method with a similar mechanism to displacement reactions. Through the reaction between molten ZnCl_2_ and the Al element in the MAX phase precursor (such as Ti_3_AlC_2_), a new Zn-based MAX phase (such as Ti_3_ZnC_2_) was synthesized. As shown in the XRD diagrams of Ti_3_AlC_2_ and Ti_3_ZnC_2_ in Figure 7b and the EDS diagram of Ti_3_ZnC_2_ in Figure 7c, compared with Ti_3_AlC_2_, the XRD peaks of Ti_3_ZnC_2_ shift to lower angles, indicating that Zn atoms have successfully replaced Al atoms. Interestingly, at 550 °C, an excessive amount of molten ZnCl_2_ salt will react with the MAX phase to synthesize MXene terminated with -Cl or without terminal groups on the surface, pioneering the creation of a fluorine-free, green, efficient MXene with only -Cl terminal groups. However, according to the obtained EDS of Ti_3_C_2_Cl_2_ (Figure 7d), it shows that the elemental composition of Ti_3_C_2_Cl_2_ still contains a small amount of Zn (0.7 atomic%), Al (2.9 atomic%), and O (6.3 atomic%). This is because the -Cl terminal group can strongly bond to the MXene surface, but when there are steps such as the presence of oxygen-containing compounds or water washing, the -Cl terminal group is less competitive than the -O terminal group and is thus replaced. This is a problem that needs to be noted and avoided when using Lewis molten salt etching.

Li et al. further summarized and explored the Lewis molten salt etching method, and the schematic diagram of its preparation process is shown in Figure 7a [86]. The principle that the A atom layer can undergo substitution with the molten salt lies in the difference in their redox potentials. Transition metal halides can act as electron acceptors to react with the A layer in the molten state. The Lewis acid molten salt has a higher redox potential compared to the MAX phase, so it can etch the MAX phase. For example, in the molten CuCl_2_ salt at 700 °C, the redox potential of Cu/Cu^2+^ is −0.43 eV, which is greater than that of Si/Si^4+^ (−1.38 eV). Therefore, the molten Cu^2+^ can oxidize the Si atoms in the MAX phase into Si^4+^, which then enters the molten salt and combines with Cl^−^ to form SiCl_4_, while Cu^2+^ is reduced to Cu atoms and remains outside the MAX phase. Then, the Cu element is removed by APS ((NH_4_)_2_S_2_O_8_). Finally, Cl^−^, Br^−^, I^−^, etc., in the molten salt combine with the M transition metal atoms, and depending on the type of molten salt, MXene with corresponding surface terminal groups can be obtained. However, the binding ability is generally weak, and subsequent substitution of functional groups can be carried out to flexibly adjust the surface functional groups, thereby obtaining MXene with other terminal groups or even without terminal groups. However, affected by the synthesis route and changes in surface groups, it often leads to a significant reduction in the rate of exfoliation and delamination of multi-layer MXene, making it difficult to obtain good electrochemical properties. Liu et al. successfully etched Ti_3_AlC_2_ using a mixed molten salt of KCl/CuCl_2_/NaCl at 680 °C in an argon atmosphere [87]. Subsequently, by inserting the organic molecule TBAOH (tetrabutylammonium hydroxide) and performing ultrasonic treatment, they successfully prepared MXene with fewer layers and improved electrochemical properties through the Lewis molten salt method. The etching and exfoliation processes of the Lewis acid molten salt method are illustrated in Figure 7e. The HF-MXenes obtained by traditional hydrofluoric acid etching usually use DMSO solvent as an intercalating agent for separation because of the matching of surface energy between DMSO and HF-MXene. However, the Ti_3_C_2_T_x_ treated with DMSO in the Lewis acid molten salt etching method is thicker and has more layers (as shown in Figure 7h), while the multi-layer MXene treated with TBAOH can be further exfoliated. The SEM images before and after exfoliation are displayed in Figure 7g. The Lewis molten salt etching is safer and more environmentally friendly compared to traditional etching methods and has the advantage of being able to regulate terminal functional groups. However, it is often difficult to obtain single-layer MXene through this redox reaction, and it is affected by the properties of the Lewis molten salt.

It is worth noting that in the past decade or so, most of the MXenes prepared in research have been derived from MAX phases. However, this process is time-consuming and complex, involving multiple steps such as the preparation of MAX, the etching from MAX to MXene, and the removal of metal impurities. To optimize this process, researchers have devoted a great deal of effort. Besides the bottom-up CVD deposition method, recently, Liu et al. proposed an innovative one-pot molten salt electrochemical etching method [81]. The specific process (Figure 7i) is divided into two processes: obtaining the MAX phase and etching the MAX phase to obtain MXene. First, an electrochemical cell containing a mixture of LiCl and KCl is heated to a stable molten state. Molten aluminum (melting point 660 °C) will wrap around the metal Ti (melting point 1668 °C) and form Ti-Al intermetallic compounds on the surface of Ti. Meanwhile, carbon nanotubes (CNTs) and reduced graphene oxide (rGO) are used as carbon sources to make TiC grow on the graphite side. Through the bottom-up growth mode, TiAlC MAX phase nuclei are generated on the carbon surface and continue to grow as TiC and Ti-Al intermetallic compounds are continuously consumed, and based on the structural framework of carbon as a template, TiAlC particles are obtained in a short time. Immediately after that, the temperature of the molten salt electrode is changed to 500 °C, and Ti_2_CCl_2_ MXene is obtained by electrochemically etching away the aluminum atom layer. The use of different carbon sources also has a significant impact on the morphology of the precursor and MXene. As shown in Figure 7j, both the Ti_2_AlC MAX phase and Ti_2_CCl_2_ MXene grown from different carbon sources retain the characteristics of CNT and GO. Compared with other methods, this method only requires a lower temperature and a shorter reaction time, greatly simplifying the preparation process.

### 3.5. Chemical Vapor Deposition (CVD) Method

**Figure 8:**
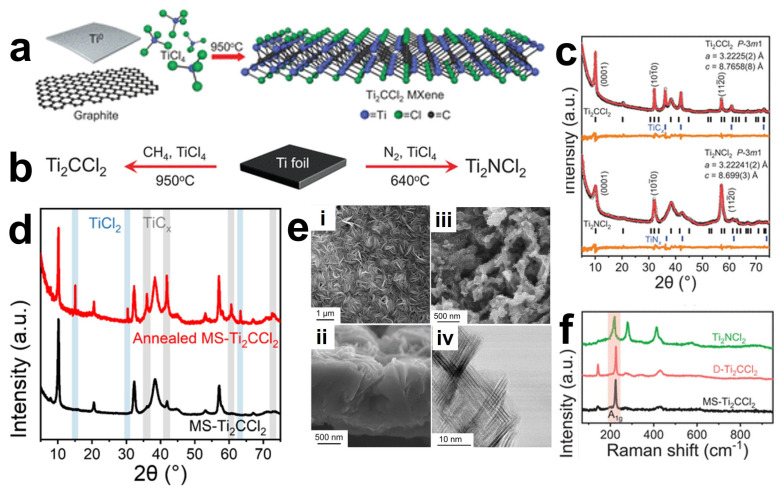
(**a**,**b**) Schematic of bottom-up direct synthesis of Ti_2_CCl_2_. (**c**) XRD patterns of CVD-Ti_2_CCl_2_ and CVD-Ti_2_NCl_2_. (**d**) Powder XRD pattern of the product of pure TiCCl MXene annealed at 950 °C for 1 day under vacuum. MXene partially decomposed into TiCl and TiC. XRD patterns of the final mixture were similar to that of DS-Ti_2_CCl_2_. (**e**) Front (**i**) and cross-section (**ii**) SEM images of CVD-Ti_2_CCl_2_. (**iii**) SEM image and (**iv**) STEM image showing that CVD-TiNCl exhibits a braided structure. (**f**) Raman spectra of CVD-Ti_2_CCl_2_ and CVD-Ti_2_NCl_2_ MXenes. [90]. Copyright 2023, American Association for the Advancement of Science (AAAS).

In the process of preparing MXenes, traditional methods such as the Lewis acid molten salt method or chemical and electrochemical etching require the use of harmful HF or energy-consuming Lewis acid molten salt. Chemical Vapor Deposition (CVD) is a typical bottom-up synthesis approach that operates at atomic/molecular scales, enabling direct growth of large-area two-dimensional materials [88]. An investigation has demonstrated the CVD synthesis of high-quality two-dimensional ultrathin Mo_2_C crystals, using methane as the carbon source at temperatures exceeding 1085 °C on layered Mo/Cu foil substrates [89]. Halim et al. [73] successfully synthesized Ti_3_AlC_2_ thin films via direct current (DC) magnetron sputtering using elemental Ti, Al, and C targets. Subsequent selective etching of the aluminum layers in aqueous HF or NH_4_HF_2_ solutions yielded Ti_3_C_2_ MXene films with a controlled thickness of approximately 19 nm. However, conventional CVD or magnetron sputtering techniques can only synthesize a limited number of MXene and strictly depend on specific substrates (such as Cu foils), failing to achieve controllable synthesis of termination-enriched MXene like Ti_2_CCl_2_ and Zr_2_CBr_2_. Wang et al. [90] reported a novel synthetic route by utilizing direct reactions between metal/metal halides and carbon/nitrogen sources without MAX phase precursors. As a representative case, Ti_2_CCl_2_ MXene was successfully grown on Ti substrates at 950 °C under an argon atmosphere using CH_4_ and TiCl_4_ as precursor gases, with the detailed synthesis process illustrated in Figure 8a,b. Through the structural analysis by XRD (Figure 8c,d) and Raman spectroscopy analysis (Figure 8f), it is revealed that the high-purity Ti_2_CCl_2_ MXene phase has been successfully prepared. Scanning electron microscopy (SEM) images showed a completely new growth morphology that dramatically diverges from conventionally synthesized counterparts. Wrinkled Ti_2_CCl_2_ MXene layers were observed to coat the substrate (Figure 8e), exhibiting vertically oriented growth to form carpet-like or spherical vesicular architectures. These nanosheets could be exfoliated into monodisperse colloidal suspensions. Notably, nitride-based MXenes may undergo dissolution in HF solutions due to insufficient chemical stability. In contrast, CVD at 640 °C using TiCl_4_ and N_2_ reactants on Ti foils enables the successful synthesis of pure-phase Ti_2_NCl_2_ MXene that is challenging to obtain through conventional approaches. Generally, directly synthesized MXenes (DS-MXenes) exhibit superior structural integrity with near-ideal stoichiometric coverage of chlorine terminations. The vertically aligned architecture achieved through CVD growth facilitates enhanced ion intercalation kinetics, resulting in exceptional rate capability in electrochemical applications.

Thickness, morphology, and size of MXene is more controllable by this method. However, two critical challenges persist in the CVD synthesis of MXenes: the nucleation mechanisms and kinetic competitions remain poorly understood, precluding precise control over crystal dimensions and defect densities; and the validated precursor combinations are currently limited to select metal halides (TiCl_4_, ZrCl_4_) with carbon/nitrogen sources (CH_4_, N_2_), restricting exploration of alternative transition metals and functional surface terminations. Future research should focus on expanding the metal repertoire (V, Nb et al.) and surface functionalization, elucidating nucleation mechanisms and optimizing synthetic protocols, and achieving wafer-scale uniform synthesis for industrial-scale production. These advancements will facilitate practical applications in high-power energy storage devices, efficient catalysts, and flexible electronics, enabling transformative industrial breakthroughs.

## 4. Rechargeable Battery

With the global demand for sustainable and efficient energy systems, the limitations of traditional electrode materials in terms of energy density, cycle life, cost of use, and environmental impact are becoming more and more prominent [91]. In recent years, nanocomposite structures based on carbon materials, transition metal oxides or hydroxides, and conductive polymers have been extensively investigated with a focus on three key parameters: surface area, electrical conductivity, and pore structure of the electrode materials. However, outstanding issues such as limited specific capacitance, low conductivity, structural degradation, slow kinetics of redox reactions, and restricted ion and electron transport remain to be addressed [92]. Therefore, the development of new electrode materials and the search for efficient, safe, and sustainable electrochemical energy conversion and storage technologies is one of the hot spots in current research.

MXene materials have a unique two-dimensional structure, excellent physicochemical properties, outstanding electrical conductivity, high specific surface area, and high chemical stability. They are considered as revolutionary materials for batteries, supercapacitors, and various electrocatalytic reactions [93,94,95]. The larger layer spacing of MXenes can allow for larger ion embedding/de-embedding, which can enhance ion diffusion and storage capacity. At the same time, this layered structure helps maintain structural stability during cycling, reducing electrode degradation and extending battery life. In recent years, the electrochemical properties of MXenes have been rapidly studied and a large amount of related literature has been published, but there is a lack of systematic organization of the research results in recent years [96]. Therefore, this review aims to fill this gap by providing a comprehensive framework to understand and utilize the potential of MXene materials for electrochemical applications, while revealing key scientific issues and future research directions.

### 4.1. Lithium–Ion Batteries

A lithium–ion battery is a high-performance secondary battery, relying on the movement of lithium ions between the positive and negative electrodes to achieve energy storage and release. Because of its high energy density, long life, and low self-discharge rate, it is widely used in the power supply system of all kinds of electronic equipment and grid energy storage [9,97,98]. This section focuses on summarizing the composite modes and structures of commonly used active electrode materials other than graphite-based anode materials with MXenes.

In order to fully utilize the unique properties of MXene materials and the anode materials, researchers have explored the use of a variety of composite methods. For example, physical mixing mechanical ball-milling mixing, wet dispersion freeze-drying mixing, the use of chemical methods, through the chemical vapor deposition on the surface of MXene in situ growth of anode materials, or the solvent thermal method of direct chemical synthesis of composite structures. Self-assembly of MXene and anode materials into multi-layer composite membranes can be realized by electrostatic interaction or other intermolecular forces. Alternatively, a specific functional group ligand can be designed to spontaneously self-assemble with MXene and the anode material to form a strong transition layer as well as a heterogeneous structure connected by covalent bonds.

**Figure 9:**
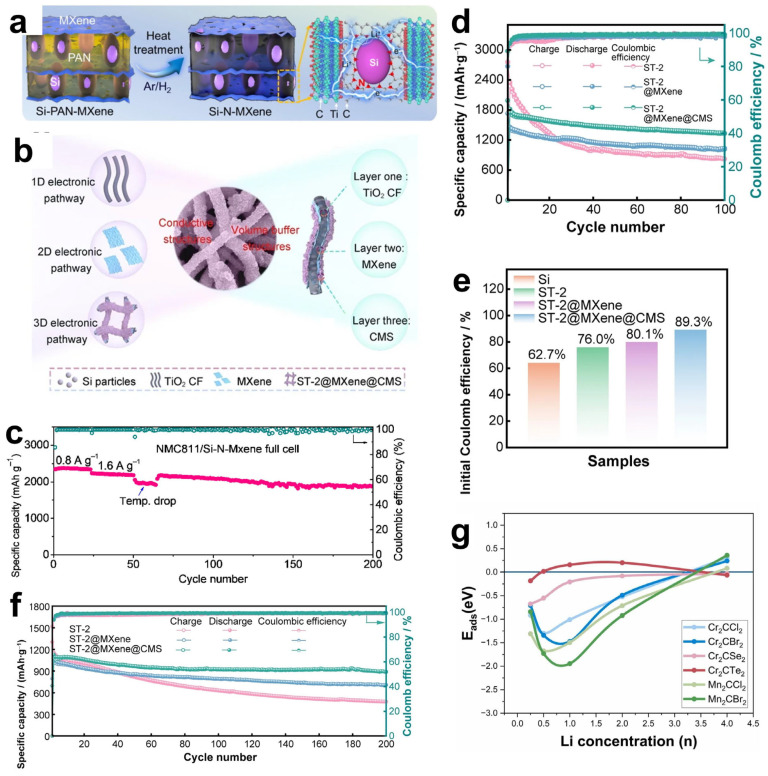
(**a**) Schematic diagram of the synthesis process for the interfacial nitrogen layer between Si and MXene. Reproduced with permission [106]. Copyright 2022, Elsevier BV. (**b**) Schematic illustration of the cycling process of the ST2@MXene@CMS electrode. Reproduced with permission [104]. Copyright 2024, Springer Nature. (**c**) Long-life cycling performance of the NMC811/Si-N-MXene full battery. Reproduced with permission [106]. Copyright 2022, Elsevier BV. (**e**) Initial charge–discharge curves and bar chart of initial Coulombic efficiency for the ST2@MXene@CMS electrode. (**d**,**f**) Cycling performance curves of the electrode at current densities of 0.1 A g^−1^ and 1.0 A g^−1^. An arrow pointing in the direction of an increasing number of cycles indicates that the material will continue the current trend in subsequent cycles. (**d**–**f**) Reproduced with permission [104]. Copyright 2024, Springer Nature. (**g**) Trends in lithium adsorption energy with lithium concentration on Cr_2_CCl_2_, Cr_2_CBr_2_, Cr_2_CSe_2_, Cr_2_CTe_2_, Mn_2_CCl_2_, and Mn_2_CBr_2_ nanosheets. Reproduced with permission [107]. Copyright 2024, Elsevier.

Graphite is the most commonly used anode material for lithium–ion batteries. However, due to the relatively low theoretical specific capacity of the graphite anode (usually 372 mAh g^−1^) [99], currently, the development of graphite electrodes has reached its theoretical limit, but it still cannot meet the requirements of technological progress for energy storage. Therefore, the search for new electrode materials is imminent. Studies have shown that in addition to graphite-based materials, silicon-based materials also have relatively high theoretical capacities [100]. Replacing the graphite anode with silicon not only effectively addresses the safety issues of the liquid electrolyte in lithium–ion batteries but also significantly improves the specific capacity of the batteries. It is regarded as one of the most promising electrode materials for next-generation high-energy lithium–ion batteries [101]. However, silicon electrodes will have a large volume change during the charge/discharge process, and this change will destroy the inherent layered structure of the electrodes, or the electrodes may even be crushed and pulverized, resulting in serious capacity loss. In order to solve the problem of volume change, the usual method is through the composite of certain conductive materials, and the formation of a multiple buffer structure or with multi-component composite, so as to inhibit the volume expansion of the electrode, enhance the mechanical stability and electrical conductivity, and effectively improve the cycling stability of the material [102]. Luckily, MXene not only has excellent mechanical properties and excellent electrical conductivity, its rich surface functional groups and layered structure can be more easily combined with the electrode material, so it is widely used as a substrate material and potential anode material composite [103,104,105]. Han et al. [106] designed a Si-N-MXene composite anode material through interface nitrogen engineering. They mixed a certain proportion of micron-sized Si powder, PAN (polyacrylonitrile), and Ti_3_C_2_T_x_ MXene and then cast the mixture onto a copper foil substrate followed by heat treatment. During the heat treatment process, PAN was converted into amorphous carbon that fixed Si and MXene, as shown in Figure 9a. Moreover, a chemical bond of N was formed to connect them, creating a solid interface nitrogen layer structure on the surfaces of Si and MXene, which could effectively enhance the adhesion between Si and MXene, thereby improving the mechanical properties. The structure of MXene creates a robust volumetric buffer layer to resist volume expansion of the silicon electrode. Si-N-MXene was used as the composite film electrode, lithium metal as the counter electrode, and 1M LiPF_6_ as the carbonate electrolyte to form a full cell. The capacity retention was 80.5% after 200 cycles (Figure 9c). The initial capacity and the capacity after 100 cycles are much larger than that of the single Si-C electrode. In solving the problem of the volume expansion phenomenon produced by Si during charging and discharging, Du et al. [104] have designed another special core-shell structure, as shown in Figure 9b. Si particles were confined in TiO_2_ carbon fibers by electrostatic spinning to obtain Si-x/TiO_2_ carbon fibers (ST-2), and ST-2 was successfully wrapped in Ti_3_C_2_T_x_ MXene under self-assembly. Then the cobalt and molybdenum salts were uniformly adsorbed on the surface of MXene in solution and then heated, and the hierarchical ST-2@Ti_3_C_2_T_x_ MXene@Co-MoS_2_ (CMS) fibers were successfully obtained, which presented a 3D electrode structure with interleaved composition. Utilizing titanium dioxide, which has a very low volume expansion rate, and MXene, which has high electrical conductivity, high specific capacity, and multiple active sites, a hierarchical and robust volumetric buffer layer was constructed together with CMS, which improves the volumetric change of Si particles in the core during charging and discharging. Through electrochemical performance testing, the ST-2@MXene@CMS electrode exhibited relatively excellent electrochemical performance. As shown in Figure 9d,f, its initial charging and discharging capacities reached 1849.9/2071.8 mAh g^−1^, and the initial Coulomb efficiency reached 89.3%, indicating a high effective utilization rate of the battery during the first charging and discharging. Moreover, at a high current density of 1.0 A/g, the reversible specific capacity reached 1333.1 mAh g^−1^ after 100 cycles. And there was still a reversible specific capacity of 891.3 mAh g^−1^ after 200 cycles. In contrast, the initial charging capacity of the pure ST-2 electrode was only 1518.9 m Ah g^−1^. And the reversible specific capacity decreased to 478.7 mAh g^−1^ after 200 cycles at 1.0 A g^−1^. This was mainly due to the limited buffering capacity of monolayer ST-2 and the structural rupture caused by the expansion of Si particles. The ST-2@MXene@CMS electrode was demonstrated to have great potential for high-energy storage, and the important role of MXenes in maintaining structural stability during their long-term cycling was also shown.

In addition, the influence of MXene surface functional groups on the energy storage properties has been extensively studied. Different groups affect the adsorption capacity by changing the binding energy and bond length of Li^+^ to MXene. Kim et al. [107] optimized the structure of M_2_CT_X_ using density functional theory (DFT) to calculate the lattice parameters and density of states (DOS), focusing on the effect of surface groups on lithium adsorption, diffusion barriers, and storage capacity, as shown in Figure 9g. For small-sized, highly electronegative groups (-Cl, Br), which form short bond lengths (2.21–2.35 Å) and low adsorption energies (−1.31~−0.71 eV) with Li^+^, the electrons are highly delocalized and the adsorption stability is strong. Small groups (-Cl) diffuse faster due to low spatial site resistance. And the large groups (-Se, Te) still keep low potential barriers through the electron leaving domain effect. However, for the lithium storage performance, the capacity of the (-Cl, Br) group system is 190–287 mAh g^−1^ in monolayer adsorption, and the -Se group system (Cr_2_CSe_2_) achieves 391.3 mAh g^−1^ by bilayer adsorption. This is due to the fact that, at high concentrations, the -Cl, Br groups are susceptible to destabilization due to Li^+^ repulsion, whereas the Se- groups maintain stability by virtue of moderate electron delocalization (adsorption energy −0.033 eV). Overall, the surface groups are regulated by “adsorption stability-diffusion efficiency-storage capacity”, which determines the actual performance of MXene as an electrode. Future research can focus on the optimization of group combination to accelerate the translation of MXene from theory to practical application.

**Figure 10:**
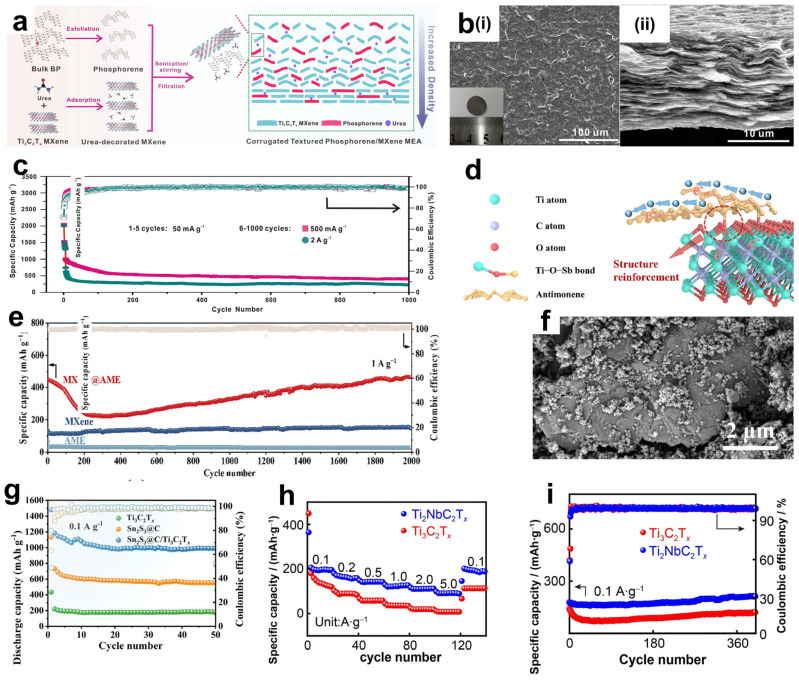
(**a**) Schematic illustration of the preparation process for corrugated phosphorene/Ti_3_C_2_T_x_ nanocomposites. (**b**) Typical corrugated patterns; (**i**,**ii**) SEM images of the surface and cross-sectional morphology of the as-prepared phosphorene/Ti_3_C_2_T_x_, respectively. (**c**) Long-term cycling stability and corresponding Coulombic efficiency of the phosphorene/MXene electrode (1–5 cycles: 50 mA g^−1^; 6–1000 cycles: 500 mA g^−1^ and 2 A g^−1^). (**a**–**c**) Reproduced with permission [110]. Copyright 2024, Springer Nature. (**d**) Schematic diagram of the structural features of the MXene@AME heterostructure. The circles in the figure indicate titanium Ti-O-Sb bonds. (**e**) Cycling performance curves of MXene@AME, MXene, and AME at 1 A g^−1^ over 2000 cycles. (**d**,**e**) Reproduced with permission [111]. Copyright 2024, Elsevier BV. (**f**) SEM image of Sn_2_S_3_@C/Ti_3_C_2_T_x_. (**g**) Cycling performance of Ti_3_C_2_T_x_, Sn_2_S_3_@C, and Sn_2_S_3_@C/Ti_3_C_2_T_x_ at 0.1 A g^−1^. (**f**,**g**) Reproduced with permission [115]. Copyright 2024, Springer Nature. (**h**) Rate capability of Ti_2_NbC_2_T_x_ and Ti_3_C_2_T_x_; (**i**) Cycling performance of Ti_2_NbC_2_T_x_ and Ti_3_C_2_T_x_ at 0.1 A g^−1^. (**h**,**i**) Reproduced with permission [117]. Copyright 2022, Springer Nature.

Phosphorus also has an excellent theoretical capacity and high electrical conductivity. Even the average discharge potential of phosphorus is higher than that of silicon, making it one of the most promising anode materials for lithium–ion batteries [108]. However, similar to silicon, phosphorus electrodes also have relatively large volume changes and slow kinetics. Research has already shown that through the liquid-phase exfoliation method, bulk black phosphorus can be exfoliated into phosphorene or black phosphorus quantum dots. The two-dimensional phosphorene has a very small volume change during the charging and discharging process, which can effectively avoid volume changes and form more lithium–ion channels, thereby significantly improving the electrochemical performance [109]. Based on this, Li et al. [110] developed a corrugated phosphorene/MXene heteronanocomposite electrode. The preparation process is shown in Figure 10a. After fully mixing and dispersing urea and Ti_3_C_2_T_x_ MXene, the mixture was dropped into a diphosphoric acid solution. In this regard, phosphorus is able to form stable P-O-Ti covalent bonds with -OH on the surface of Ti_3_C_2_T_x_ MXene, thereby making a membrane after self-assembly. The incorporation of polar urea molecules can avoid the concentration of phosphorus aggregating on the active sites on the MXene surface and promote a uniform distribution among the MXene lamellae, such as the corrugated protrusions in Figure 10b(i). In addition, the typical accordion-like lamellar structure of MXene is retained (Figure 10b(ii)). Through bridging and crosslinking, phosphene and MXene form stable ionic or charged 2D lamellar corrugated heterostructures. When electrons transition from the Fermi level of MXene to the Fermi level of phosphorene, a migration of the work function from a higher value to a lower value occurs, aligning the Fermi levels of the two materials. Moreover, changes in the electron concentration and vacancy concentration will occur at the heterostructure. These changes can improve the charge conductivity, reduce the energy barrier for the migration of the solid electrolyte interface (SEI) film of lithium ions on the electrode surface, and greatly enhance the electrochemical performance. The formed conductive framework can significantly increase the stability of the structure and volume changes. Electrochemical performance analysis was performed on a half-cell system consisting of a phosphorene/MXene nanocomposite film as the anode and a 1 M LiPF_6_-based carbonate mixed electrolyte solution, as shown in Figure 10c. At a current density of 100 mA g^−1^, the electrode delivers an ideal capacity of 1463.2 mAh g^−1^ and maintains a favorable reversible capacity of 848.3 mAh g^−1^ after 100 cycles. This demonstrates excellent cycling stability, which significantly surpasses that of pure MXene and phosphorene electrodes. All these data show its great potential as a new anode material for lithium–ion batteries. Antimony (Sb), similar to phosphorus, also has a unique monolayer two-dimensional structure, antimonene. Antimony also has high theoretical capacity with high lithium insertion potential. However, it has the same problems of large volume change and poor electronic conductivity during charging and discharging. Bo et al. [111] counted and synthesized covalently bonded Ti_3_C_2_T_x_ MXene@antimonene (MXene@AME) heteroanodes. Antimonene is self-assembled with the -OH groups on the surface of MXene, which serves as both a conductive matrix and a stress-absorbing layer. A strong heterostructure (Ti-O-Sb) is formed between the two under electrostatic action, schematically shown in Figure 10d. Similar to phosphene, it can greatly improve electrochemical performance. The MXene@AME anode exhibits outstanding cycling performance at both a lower current density of 0.2 A g^−1^ and a higher current density of 1 A g^−1^. As shown in Figure 10e, after 300 cycles at the low current density, it can still maintain a capacity of 455 mAh g^−1^, with a retention rate as high as 93.8%. The phenomenon that the capacity first decreases and then recovers, and is even higher than the initial capacity (447 mAh g^−1^), may be due to the formation of an unstable SEI layer in the initial charging and discharging cycles, the incomplete activation of the electrode material, or the degradation of the electrode structure. Subsequently, it is because of the stable reconstruction of the Ti-O-Sb bond and the formation of a stable SEI layer. In contrast, the reversible capacity of pure Ti_3_C_2_T_x_ MXene was only 81.7 mAh g^−1^ after 100 cycles under the same conditions. This may be attributed to the reduction of active sites due to the stacking of MXene layers, resulting in a faster capacity decay during cycling.

In addition, alloy-based anode materials also have high lithium storage capacity through alloying and de-alloying mechanisms [112,113]. But there are also problems such as poor electrical conductivity during charging and discharging, large volume changes, and dendrite growth during lithium–ion deposition [114]. Tin-based materials, featuring high theoretical specific capacity and energy density, have also attracted widespread attention. Guan et al. [115] synthesized a Sn_2_S_3_@C/Ti_3_C_2_T_x_ composite electrode material with a laminated leaf-like structure. Sn_2_S_3_ was uniformly dispersed on several layers of MXene nanosheets after being coated by nanosized C, as shown in Figure 10f. Interestingly, the mechanical ball-milling process employed in the preparation introduces certain defects on the surface of Ti_3_C_2_T_x_. These defects facilitate the stable anchoring of Sn_2_S_3_@C onto the MXene surface and effectively mitigate the internal stress generated during charge–discharge cycles, thereby enhancing the overall stability of the composite structure. When Sn_2_S_3_@C/Ti_3_C_2_T_x_ composites were used as an anode for lithium–ion batteries (Figure 10g), the reversible specific capacity was 989.6 mAh g^−1^ after 50 cycles at a current density of 0.1 A g^−1^. Cheng et al. [116] employed a solvothermal method, adsorbing tin ions on the MXene surface to induce in situ nucleation and growth, and successfully prepared Sn-C bonded MXene/SnSe_2_ composite electrode materials. SnSe_2_ nanosheets vertically and completely cover the MXene surface, forming a laminar structure. However, electrodes of this structure are not stable under the action of high-density currents. Scanning electron microscopy (SEM) observations show that after 100 cycles at a current density of 2000 mA g^−1^, SnSe_2_ nanosheets undergo in situ fragmentation into nanoparticles that disperse between MXene layers. This structural evolution leads to a notable increase in battery capacity and enhancement in electrochemical performance. The underlying mechanism involves the fragmented particle structure significantly expanding the specific surface area of the electrode material, which not only enables more lithium ions to insert into the material but also provides abundant ion transport pathways. These pathways accelerate lithium–ion diffusion rates, thereby contributing to the capacity improvement.

In all of the above, a composite electrode is formed from a single-transition layer of metal MXene. It has been shown that a variety of M-metals in MXenes can effectively improve the stability of the structure during cycling [118,119]. Liu et al. [117] prepared a double-transition metal Ti_2_NbC_2_T_x_ MXene and compared its electrochemical performance as an anode with that of the single-transition metal Ti_3_C_2_T_x_ MXene, as shown in Figure 10h,i. At 0.1 Ag^−1^ current density, the Ti_2_NbC_2_T_x_ MXene electrode provides a discharge capacity of 305.7 mAhg^−1^ at the first cycle which is less than that of Ti_3_C_2_T_x_ MXene. However, Ti_2_NbC_2_T_x_ anodes consistently provide superior multiplication performance even at different current densities. And after 4000 cycles, the capacity of Ti_2_NbC_2_T_x_ was 198 mAh g^−1^ with a retention rate of 81%, which was much higher than that of Ti_3_C_2_T_x_ anode (47.4%), and the cycling efficiency was always higher. The reason may be due to the fact that the addition of Nb enlarges the Ti_2_NbC_2_T_x_ MXene interplanar spacing and provides more active sites, resulting in a lower charge transfer resistance and a faster Li^+^ migration rate. Lu et al. [120] used self-assembly to anchor zero-dimensional carbon dots (CDs) on the Ti_2_NbC_2_T_x_ MXene surface via Ti(Nb)-O-C covalent bonds to successfully form a composite anode. After 10,000 cycles at 5 A g^−1^, its lithium storage capacity was able to remain stable at 217.3 mAh g^−1^. Compared with Ti_3_C_2_T_x_ and Ti_2_NbC_2_T_x_, the Ti_2_NbC_2_T_x_@CDs exhibit higher specific capacity and stronger stability. This is attributed to the synergistic effect of dual transition metals in Ti_2_NbC_2_T_x_, which expands the interlayer spacing and optimizes the electronic structure, thereby effectively enhancing ion diffusion rates and charge transfer efficiency. Additionally, the double-transition metal MXene demonstrates significantly superior lithium storage capacity, cycle life, and rate performance compared to single-metal MXenes, highlighting its greater application potential in electrochemical energy storage systems.

Overall, the current mainstream strategy for constructing high-performance 2D MXene-based electrodes typically involves combining MXene nanosheets with other electrochemically active phases (such as metal hydroxides, transition metal oxides/sulfides) to construct diverse 3D architectures. This is because these electrochemically active phases, owing to their unique electronic structures and variable valence states, can undergo reversible transformations during lithium intercalation—for example, titanium-based and vanadium-based oxides can reversibly convert to provide high-capacity lithium storage sites, thereby enabling efficient charge transfer in electrochemical processes. Moreover, constructing 3D MXene electrodes with structures like three-dimensional porous structures, three-dimensional columnar structures, three-dimensional stacked structures, and three-dimensional skeleton structures can effectively prevent the restacking of two-dimensional nanosheets due to van der Waals forces.

It is worth noting that the ST-2@MXene@CMS electrode demonstrated high specific capacity and cycling stability, but its initial Coulombic efficiency (89.3%) still has room for improvement. This reflects the fact that the problem of controlling SEI films on silicon surfaces has not yet been fully solved. Combined with the nitrogen interface layer design by Han et al. [106], it can be speculated that the uniformity of the interface chemical composition is the breakthrough direction for future electrochemical performance. The introduction of nitrogen not only enhances the interfacial binding force but may also suppress the excessive decomposition of the electrolyte by adjusting the electron density on the silicon surface. With the assistance of machine learning for structural optimization, precise control of the electrode porosity and the distribution of the conductive network can be achieved, thus overcoming the limitation of “the complexity of ion diffusion paths within the three-dimensional structure”. In addition, current research has not fully utilized the interlayer active sites. The lithium–ion storage capacity between phosphorene layers can be further activated through defect engineering (such as phosphorus vacancies and antimony doping). Inspired by the corrugated design of Li et al. [110], if nanoscale support columns or functional molecules like conductive polymers are introduced between the layers, a dual-site storage mechanism of surface adsorption and interlayer insertion can be established while maintaining the interlayer distance, so that the specific capacity of two-dimensional materials is no longer limited by the specific surface area.

### 4.2. Sodium–Ion Batteries

Lithium is a very small element in the earth’s crust, and the cost of using lithium–ion batteries has increased dramatically in recent years as the scale of development and use has expanded. As a result, energy storage technologies other than lithium are attracting much attention [121]. While sodium is extremely abundant in the earth, making it one of the most promising alternatives to lithium–ion batteries [122]. However, the ionic radius of sodium is larger than that of lithium, resulting in more difficult intercalation and deintercalation of sodium ions compared to lithium ions, along with slower kinetics. As a result, traditional anode materials for lithium ions, such as graphite, are not suitable for sodium ions [123,124]. Therefore, the development of anode materials with high sodium storage capacity and long life is the current problem to be solved for sodium–ion batteries. Density Functional Theory (DFT) calculations show that sodium ions (Na^+^) can be smoothly embedded into the lattice structure of Ti_3_C_2_T_x_ MXene with a low diffusion energy barrier of 0.41 eV [125]. It is shown that Ti_3_C_2_T_x_ MXene is expected to achieve efficient charge transfer and storage when used as an anode material for sodium–ion batteries.

**Figure 11:**
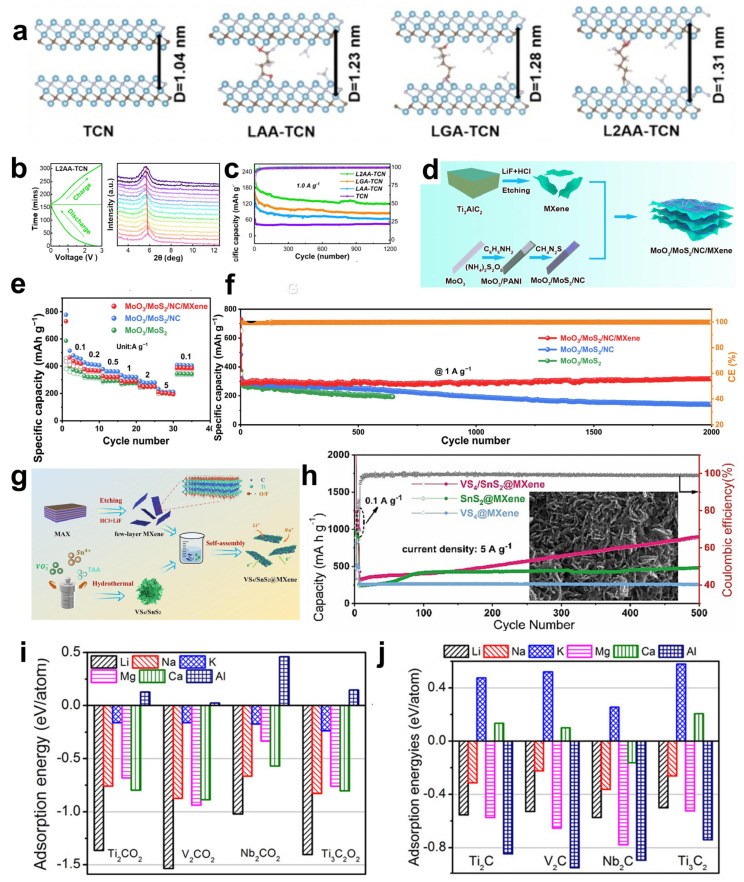
(**a**) Schematic diagrams of three LXA-TCN structures. (**b**) Galvanostatic charge–discharge (GCD) curves and in situ XRD patterns of L_2_AA-TCN. (**c**) Cycling stability of LXA-TCN and TCN at 1.0 A g^−1^. (**a**–**c**) Reproduced with permission [128]. Copyright 2024, Elsevier BV. (**d**) Synthesis process and structural schematic of the MoO_3_/MoS_2_/NC/MXene composite. (**e**) Specific capacities of different samples at various current densities. (**f**) Cycling performance curve of MoO_3_/MoS_2_/NC/MXene at 1 A g^−1^. (**d**,**f**) Reproduced with permission [132]. Copyright 2024, Elsevier BV. (**g**) Specific capacity of different samples at different current densities. (**h**) Cycling performance curves of MoO_3/_MoS_2_/NC/MXene at 1 A/g. (**g**,**h**) Reproduced with permission [133]. Copyright 2024, Elsevier BV. (**i**) Ion adsorption energy on -O terminated MXene nanosheets. (**j**) Ion adsorption energy on non-terminated MXene nanosheets. (**i**,**j**) Reproduced with permission [127]. Copyright 2014, American Chemical Society.

The embedding mechanism of sodium ions in MXene is important for the study of sodium–ion battery materials. During the charge–discharge processes of MXene electrodes, the two primary factors contributing the most to metal–ion capacity are solvated metal ions adsorbed on the MXene surface and completely desolvated metal ions intercalated into the interlayer spaces of MXene sheets [126]. Xie et al. [127] discovered that surface functional groups and transition metal species significantly influence the capacity and voltage of MXenes. Specifically, M_2_C MXenes (M = Sc, Ti, V, etc.) with -O terminations or no functional groups exhibit better applicability for sodium–ion batteries compared to those with other terminal groups. DFT calculations also confirm this. Sodium ions can be efficiently adsorbed by MXene without termination groups or -O terminals through good negative adsorption energy. On Ti_2_CO_2_, the adsorption energy of Na^+^ is −0.202 eV/atom (as shown in Figure 11i). While on the Ti_2_C surface, despite the absence of O atom coordination, Na^+^ can still form a stable adsorption layer through metal–carbon bond interactions with an adsorption energy of approximately −0.1 eV/atom, as depicted in Figure 11j. Meanwhile, terminal-free and -O terminated MXenes exhibit low diffusion barriers and open-circuit voltages (OCV) for sodium ions, indicating their promise as anode materials with high capacity and excellent rate performance. In contrast, -F and -OH terminated MXenes show weaker adsorption capabilities toward sodium ions. Beyond serving as electron-rich terminal groups that enhance adsorption of metal ions like Na^+^, oxygen terminations (-O) also participate in heterojunction formation through both electrostatic interactions and covalent bonding. For example, Ti_3_C_2_T_x_ MXene, with abundant -O terminations on its surface, forms Ti-O-P/Ti-O-Sb covalent bonds with phosphorene/antimonene to create heterojunctions. MXene surfaces obtained by etching Ti_3_AlC_2_ with LiF/HCl carry negative charges due to their rich -O, -OH, and -F terminations. Cationic modification (PEI) of Si/TiO_2_ carbon fibers enables the formation of stable composite structures with MXene via electrostatic bonding, leveraging the charged nature of these terminal groups.

Similar to lithium–ion batteries, the stacking of MXenes nanolayers causes their active surface to shrink, resulting in a decrease in their electrochemical performance. Moreover, the insertion and extraction of sodium ions will affect the volume changes of the anode material, causing expansion and contraction, resulting in structural instability and even cracking and pulverization. Similarly, by rationally constructing MXene-based 3D porous composite materials and taking advantage of the high electrical conductivity and good mechanical flexibility of MXene, these problems can be effectively solved. Zhang et al. [128] utilized amino acid molecules, including L-aspartic acid, L-glutamic acid, and L-2-aminoadipic acid (abbreviated as LAA, LGA, L_2_AA, collectively named LXA), which possess surface -COOH groups. These groups form strong amide (HN-C=O) bonds with the -NH_2_ groups of amino-functionalized Ti_3_CN-NH_2_ MXene. This bonding process creates a stable interlayer tenon–mortise structure in the composite. The resulting materials are named LAA-TCN, LGA-TCN, and L_2_AA-TCN, collectively abbreviated as LXA-TCN, as shown in Figure 11a. This tenon–mortise structure is analogous to pillars between nanolaminar sheets, effectively enabling controlled expansion of the interlayer spacing. In the in situ XRD pattern of L_2_AA-TCN (Figure 11b), the (002) diffraction peak exhibits negligible shifts during discharge and charge processes, indicating that L_2_AA-TCN maintains a stable lamellar structure. This pillar effect, combined with the strain tolerance of the MXene skeleton, enables stable and fast Na^+^ storage. At a current density of 1.0 A^−1^, after 1200 cycles (Figure 11c), the reversible capacities of L_2_AA-TCN, LGA-TCN, and LAA-TCN are 121.7, 84.1, and 64.1 mAh g^−1^, respectively, with capacity retentions of 68.1%, 63.5%, and 61.2%, significantly higher than that of pristine Ti_3_CN MXene (40.5%). Beyond structural tuning of MXene-based composites, transition metal oxides/sulfides, owing to their higher mass capacities, have been widely employed as electrode materials for lithium–ion batteries [129,130]. For instance, upon simple combination with graphene, MoO_3_ nanoplates serve as anode materials for sodium–ion batteries and exhibit outstanding electrochemical performance [131]. But this type of transition metal oxides/sulfides is also accompanied by the disadvantages of insufficient electrical conductivity and large volume expansion rate during charging and discharging. Yu et al. [132] employed vapor sulfidation and NC coating methods to encapsulate an N-doped carbon layer on the surface of the MoO_3_/MoS_2_ heterostructure. The resulting material was then physically mixed with MXene nanosheets and modified to form a MoO_3_/MoS_2_/NC/MXene composite, as illustrated in the process structure of Figure 11d. Due to the different electron affinity energies (Efbs) of MoO_3_ and MoS_2_, a built-in electric field is formed between MoS_2_ and MoO_3_ until they reach the same Efb. This heterogeneous heterostructure acts as an accelerator for electron transport, thereby enhancing the composite’s ultrafast electron diffusion and sodium–ion transport capabilities. The introduction of sulfidation, NC coating, and MXene significantly enhances electrical conductivity and ionic diffusion/transport kinetics. Electrochemical performance analysis (as shown in Figure 11e,f) reveals that the MoO_3_/MoS_2_/NC/MXene electrode delivers a reversible capacity of 464 mAh g^−1^ at a low current density of 0.1 A g^−1^, higher than the MXene-free MoO_3_/MoS_2_/NC electrode (326 mAh g^−1^). Notably, it retains a discharge capacity of 202 mAh g^−1^ even at a high current density of 5 A g^−1^. Additionally, at a current density of 1 A g^−1^, the electrode material retains a high discharge capacity of 320 mAh g^−1^ after 2000 cycles, with a capacity retention close to 100%, demonstrating excellent electrochemical performance. In contrast, the MXene-undoped MoO_3_/MoS_2_/NC electrode experiences a drastic capacity drop to 141 mAh g^−1^ after 2000 cycles. Clearly, without the mechanical support of MXene, the electrode structure is prone to collapse. By contrast, the introduction of MXene establishes a 2D conductive network that significantly accelerates electron transport. Meanwhile, its flexible nanosheets effectively buffer volume expansion during cycling and optimize ionic diffusion kinetics, thereby achieving synergistic enhancement of electrochemical performance.

In addition to the above-mentioned tin sulfides and MoS_2_, other 2D metal sulfides such as VS_2_ are regarded as the next generation of anode materials for lithium/sodium–ion batteries due to their unique layered structure and ultra-high theoretical specific capacity [134,135]. But they still can not avoid the volume expansion and agglomeration during the cycle process, and their own poor electrical conductivity, resulting in a decline in electrochemical performance [136]. Vanadium sulfides (e.g., VS_2_, VS_4_, and V_5_S_8_) have been used as energy storage materials due to their multivalent properties. In particular, VS_4_ has a quasi-one-dimensional chain structure connected by S_2_^2−^ dimers, and the chains are linked by weak van der Waals forces to form a wide chain spacing (5.83 Å), which facilitates Li^+^ and Na^+^ insertion and extraction [137,138]. Similarly, the weak van der Waals forces between the layers in the two-dimensional layered structure of SnS_2_ facilitate the embedding/de-embedding of ions and effectively mitigate the volume expansion of the electrode material. Hou et al. [133] uniformly fixed the VS_4_/SnS_4_ heteronanosheets obtained through hydrothermal reaction on the surface of MXenes by electrostatic self-assembly, and prepared a heterogeneous-structured bimetallic sulfide VS_4_/SnS_2_@MXene nanosheet, whose structure is shown in Figure 11g. Similarly, this heterogeneous interface accelerates the transport of ions and electrons during electrode reactions. MXene nanosheets effectively enhance the electrical conductivity of composite electrodes, suppress volume expansion, and improve the reaction kinetics of the electrode. Based on this, cycling performance tests were conducted on different electrodes (as shown in Figure 11h). The results show that after 200 cycles at 0.1 A g^−1^, the VS_4_/SnS_2_@MXene composite electrode delivers a high capacity of 1565.3 mAh g^−1^, far exceeding that of the SnS_2_@MXene and VS_4_@MXene electrodes. Even after 500 cycles, the VS_4_/SnS_2_@MXene composite electrode maintains its original lamellar structure, indicating no severe agglomeration or pulverization occurs during charging/discharging processes. Notably, the MXene-free VS_4_/SnS_2_ composite electrode only delivers a capacity of 238 mAh g^−1^. This is because the 3D structure of MXene increases the electrode’s surface area, enabling it to accommodate more VS_4_/SnS_2_ heterojunctions on its surface. This feature thereby creates abundant ion storage sites, ultimately enhancing the electrochemical performance of the composite electrode.

The ionic radius of Na^+^ (1.02 Å) is significantly larger than that of Li^+^ (0.76 Å), leading to higher lattice stress during its intercalation. Zhang et al. achieved precise regulation of interlayer spacing through covalent bonding between amino acid molecules and MXene layers, providing an adaptive space for Na^+^ insertion. In the MoO_3_/MoS_2_/NC/MXene system, Yu et al. revealed that the built-in electric field at the heterojunction, enabled by Fermi level alignment and defect synergistic effects, significantly optimizes ion migration kinetics. Hou et al. further demonstrated in their three-dimensional VS_4_/SnS_2_@MXene bimetallic composite system that the combination of nanoscale interfacial covalent bonding (Ti-O-S bond collaborative anchoring) and microscale heterogeneous porous frameworks achieves performance enhancement through multiscale synergistic regulation. However, the matching between MXene surface charge density and Na^+^ adsorption energy remains overlooked. Future research should integrate theoretical calculations and in situ characterization to precisely tune surface chemical states, such as through fluorination (-F) or nitrogen doping, to enhance Na^+^ binding capability.

### 4.3. Lithium–Sulfur Batteries

Unlike lithium and sodium–ion batteries, the charging and discharging process of Li–S batteries is carried out by breaking and forming S-S chemical bonds in the middle of the S_8_ molecule. When cathode sulfur is reduced to lithium sulfide (Li_2_S), it can theoretically provide a specific capacity of 1675 mAh g^−1^ and an energy density of 2800 Wh/Kg, which is approximately three to five times higher than that of conventional lithium–ion batteries [139]. However, the research and development of lithium–sulfur batteries is mainly hindered by three problems: (1) the low conductivity of the sulfur cathode hinders the redox reaction, resulting in a reduction in the efficiency of the use of elemental sulfur. (2) In the process of charging and discharging, soluble long-chain sulfide will be generated and pass through the diaphragm to circulate between the positive and negative electrodes, resulting in the shuttle effect to make the active sulfur loss and reduce the capacity of the battery. (3) Volume expansion during charging and discharging [140,141,142].

MXene, with its abundant surface functional groups or grafted active sites, can effectively anchor lithium polysulfides (LiPSs) and promote the formation of metal–sulfur bonds, thereby inhibiting the aforementioned shuttle effect. In 2017, Nazar’s research team systematically investigated the polysulfide anchoring mechanism of Ti-based MXene materials (including Ti_2_C, Ti_3_C_2_, and Ti_3_CN systems) for lithium–sulfur battery systems [143]. Based on density functional theory (DFT) calculations, they revealed a synergistic anchoring mechanism between the -OH terminations on the MXene surface and Ti active sites: during the electrochemical cycling process, -OH groups first form thiosulfate coordination intermediates with polysulfides, and then the exposed titanium atoms form Ti-S covalent bonds with sulfur species through their strong Lewis acidic properties.

Through density functional theory (DFT) calculations, it was found that Ti_2_CF_2_ and Ti_2_CO_2_, as conductive anchoring materials for lithium polysulfides (LiPSs), suppress the shuttle effect through distinct mechanisms [144]. The F-functionalized surface of Ti_2_CF_2_ inhibits the shuttle effect via strong interactions with LiPSs, with binding energies (1.02–1.15 eV) higher than those with electrolytes (0.74–0.79 eV), effectively anchoring LiPSs on the cathode and preventing their dissolution in the electrolyte. In contrast, the O-functionalized surface of Ti_2_CO_2_ converts soluble high-order LiPSs (Li_2_S_8_, Li_2_S_7_, Li_2_S_6_) into insoluble elemental sulfur, thereby suppressing the shuttle effect. This stage-dependent mechanism provides a theoretical basis for designing MXene-based functional separators by efficiently restraining polysulfide shuttling.

**Figure 12:**
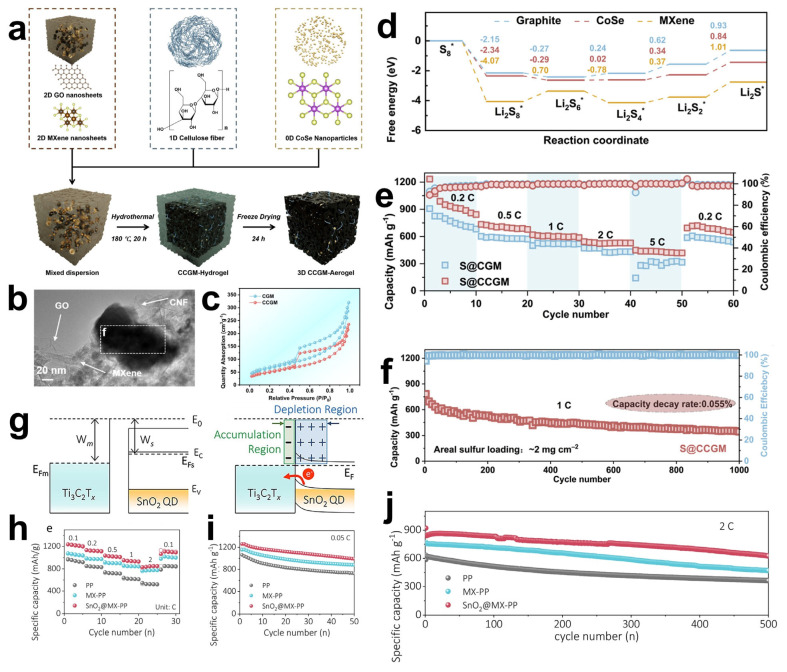
(**a**) Schematic diagram of the synthesis process of the three-dimensional CCGM aerogel. (**b**) Transmission electron microscope image of CCGM. (**c**) N_2_ adsorption–desorption isotherms of CGM and CCGM. (**d**) Relative Gibbs free energy spectra of reducing LiPSs on CoSe, graphene, and MXene substrates. The asterisk in the upper right corner represents the adsorption state in which the sulfur species adsorbed on the surface of the electrode material is in. (**e**) Rate performance of S@CGM and S@CCGM cathodes. (**f**) Long-term cycling stability of the S@CCGM electrode at a high current rate of 1C. (**a**–**f**) Reproduced with permission [26]. Copyright 2024, Elsevier BV. (**g**) Mott–Schottky type contact band diagrams of MXene (work function Wm = 4.37 eV) and SnO_2_ (work function Ws = 3.84 eV). (**h**) Rate performance of batteries with different separators at current rates from 0.1C to 2C. (**i**) Cycling performance at a small current rate of 0.05C. (**j**) Long-term cycling performance at a high current rate of 2C. (**g**–**j**) Reproduced with permission [148]. Copyright 2024, Springer Nature.

In lithium–sulfur batteries, apart from its relatively high conductivity, the catalytic effect of MXene is also of great importance. MXene is equipped with abundant surface functional groups or can be grafted with more active sites, which can effectively anchor LiPSs and promote the formation of metal-S bonds, thereby suppressing the aforementioned shuttle effect. The main strategy in past research was to introduce highly conductive porous materials as scaffolds to alleviate volume expansion and enhance the conductivity of ions/electrons, thus addressing the above-mentioned problems [145,146]. Optimizing the separator design to block the transport channels of polysulfides through physical shielding and chemical anchoring is one of the important strategies to improve the performance of lithium–sulfur batteries. Compared with traditional transition metal oxides/sulfides, transition metal selenides have a larger atomic radius and lower ionization energy. Their unique d-electron structures endow them with higher electrocatalytic performance. In particular, the CoSe catalyst can accelerate the conversion of polysulfides and Li_2_S. During the solid–solid conversion stage, it exhibits excellent adsorption energy and free energy [147]. Building on this, Han et al. [26] utilized graphene oxide as a conductive and assembly framework, and achieved the self-assembly of 0D CoSe nanoparticles, 1D carbon nanofibers (CNFs), and 2D MXene via a hydrothermal reaction, yielding a 3D aerogel functional separator (CCGM), as illustrated in Figure 12a. Transmission electron microscopy (TEM) observation of CCGM’s microscopic morphology (Figure 12b) reveals that CoSe nanoparticles (~141 nm) are uniformly anchored within a three-dimensional network composed of carbon nanofibers, lamellar MXene, and a graphene substrate. Nitrogen adsorption–desorption isotherm analysis indicates that the CCGM aerogel (Figure 12c) possesses a high Brunauer–Emmett–Teller (BET) specific surface area of approximately 174.94 m^2^/g, providing abundant active sites for the adsorption and conversion of polysulfides. Notably, in lithium–sulfur batteries, the reduction of Li_2_S_2_ to Li_2_S during discharge determines the battery’s discharge rate. The Gibbs free energy profile from S_8_ to Li_2_S in Figure 12d reveals that the Gibbs free energy of CoSe (0.84 eV) is lower than that of graphene (0.93 eV) and Ti_3_C_2_O_2_ (1.01 eV). This indicates that nanosized CoSe catalyst particles dispersed in the 3D conductive network can effectively accelerate Li2S conversion, enabling fast conversion kinetics.

Within this network, the reticular structure formed by two-dimensional graphene and MXene effectively captures polysulfides, mitigating volume expansion and the shuttle effect during charging and discharging processes. When the porous sulfur electrode integrated with CCGM aerogel serves as the sulfur cathode (Figure 12e,f), it demonstrates a high initial discharge specific capacity of 1205 mAh g^−1^. Even at a high rate of 5C, it achieves reversible conversion of polysulfides. After 300 cycles, it retains a capacity of 559.3 mAh g^−1^ with a decay rate of only 0.055% per cycle, exhibiting excellent electrochemical stability. By designing such adsorption–catalytic functional separators, the migration of polysulfides toward the anode can be restricted. Controlling permeability to enable selective Li^+^ transport retains dissolved lithium polysulfides (LiPSs) on the cathode side, effectively alleviating the shuttle effect, improving charge transfer, and inhibiting the nucleation and growth of Li dendrites.

In lithium–sulfur batteries, when the electronic structures on both sides of the heterojunction change, it can effectively enhance the strong chemical adsorption and catalytic effect on LiPSs [149]. Deng et al. [148] first obtained single-layer Ti_3_C_2_T_x_ MXene (SnO_2_@MXene) nanosheets modified by SnO_2_ nanoparticles through the hydrothermal method. Then, they mixed them with N-methyl-2-pyrrolidone and dried them in a vacuum on both sides of a polypropylene film to obtain a special heterogeneous structure interlayer (SnO_2_@MXene-modified PP film). The SnO_2_@MXene heterojunction, as shown in Figure 12g, exhibits a charge transfer behavior where electrons migrate from SnO_2_ to MXene when Ti_3_C_2_T_x_ MXene comes into contact with semiconducting SnO_2_. This process forms a negatively charged accumulation region near MXene, generating a built-in electric field (BIEF) that promotes electron transfer, thereby enhancing the adsorption and conversion of lithium polysulfides (LiPSs).

Taking CNT (carbon nanotube)/S as the cathode and using three intermediate diaphragms, namely PP, MXene-PP (single-layer MXene without SnO_2_ modification), and SnO_2_@MX-PP, to assemble coin batteries for different electrochemical performance analyses, as shown in Figure 12h–j. The SnO_2_@MXene-PP separator-based coin cell delivers a high initial capacity of 1414 mAh g^−1^ at 0.05C, and even at a high current rate of 2C, it retains an initial capacity of 845 mAh g^−1^. This value is significantly higher than those of MX-PP (1230 mAh g^−1^) and PP (1150 mAh g^−1^), with a 25.3% capacity increase especially at the second discharge plateau (Li_2_S deposition), indicating more efficient conversion of lithium polysulfides (LiPSs). Ye et al. [150] also designed a selective membrane that combines diamino crown ether (CE) with two-dimensional graphene oxide (GO) as an ion sieve layer. The modified GO-CE ion sieve layer can also be further combined with MXene to improve ionic conductivity, and the abundant functional groups brought by MXene and CE can form supramolecular channels, thereby suppressing the shuttle of Lps. This intermediate layer with a special structure has been proven to effectively improve the rate performance and cycling stability of the battery and enhance the electrocatalytic efficiency of sulfur in lithium–sulfur batteries.

To address the shuttle–deposition cycle of LiPSs, the heterojunction enhances the chemical anchoring of LiPSs through charge redistribution, while the built-in electric field accelerates the electron migration, providing a kinetic advantage for the catalytic reaction. By introducing semiconductor/metal compounds to construct heterojunctions (e.g., transition metal selenides, oxides), the electronic states on the surface of MXene can be targeted to optimize the adsorption energy and conversion pathway of LiPSs. In the future, the heterojunction components matching with MXene can be screened in combination with density functional theory (DFT) calculations to realize the precise regulation of interfacial effects.

### 4.4. Multivalent Ion Batteries

In addition to the combination of MXene as a substrate or catalytic main body with LIB mentioned above, other types of multivalent ion batteries (MVIBs) are also expected to have a relatively high theoretical capacity and are expected to become the next-generation new batteries [10,127]. The difference from the aforementioned lithium–ion and sodium–ion batteries lies in that high-valence ions (such as Zn^2+^, Mg^2+^, and even higher-valence Al^3+^, etc.) can carry more charges, thus having the potential for greater energy density and faster charging and discharging [10,151]. Research on zinc–ion batteries began as early as the 1970s. However, due to the limitations of materials at that time, it did not develop significantly. It was not until recently that MXene materials with excellent energy storage potential were discovered and the research and development of lithium–ion related batteries reached a bottleneck that the research on multivalent ion batteries such as zinc–ion batteries was explored again. Nevertheless, the most prominent problem with multivalent ion batteries lies in their limited specific capacity. Since their volume is much larger than that of lithium ions, it leads to slow insertion kinetics in the electrode materials [152]. In addition, due to the uneven zinc deposition, zinc dendrites are prone to form on the zinc anode during the charging and discharging process, and they may even pierce the electrode separator, resulting in a short circuit, which will affect the battery structure and safety [153]. Therefore, the electrode structure needs to be further optimized.

**Figure 13:**
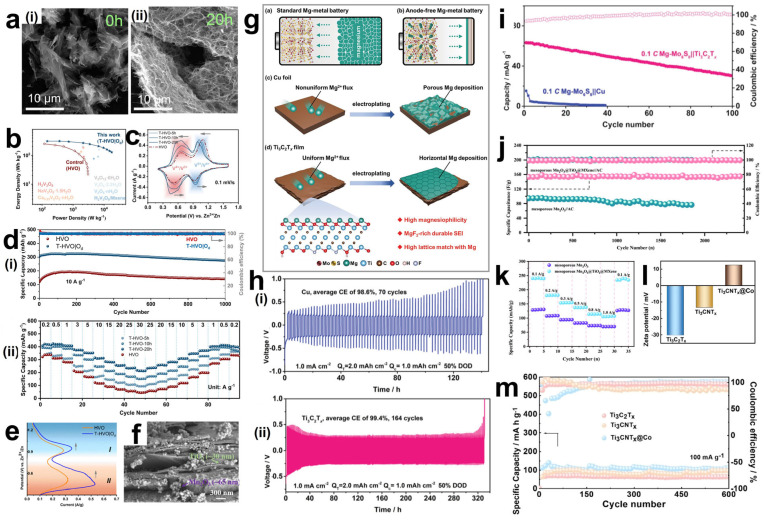
(**a**) SEM images of HVO and T-HVO with different induction growth times. HVO selectively grows on Ti_3_C_2_ MXene (**i**) as a reticulated porous structure (**ii**). (**b**) Ragone diagrams of different vanadium oxides as cathodes of zinc–ion batteries. (**c**) Comparison of CV curves of HVO and T-HVO electrodes in the fourth cycle. (**d**) Electrochemical properties of HVO and T-HVO: (**i**) Cycling stability test of HVO and T-HVO at 10 A/g. (**ii**) Comparison of rate performance at different current densities. (**e**) Current density diagrams at different potentials during the battery discharging process, showing the current response during the battery discharging process and the changes in the response diffusion coefficient at different potentials. Zone I represents the redox reaction of V^5+^/V^4+^, and Zone II represents the redox reaction of V^4+^/V^3+^. (**a**–**e**) Reproduced with permission [159]. Copyright 2024, Wiley-VCH. (**f**) SEM image of mesoporous Mn_2_O_3_@TiO_2_@MXene. Reproduced with permission [160]. Copyright 2023, Wiley-VCH. (**g**) Schematic diagrams of a standard battery with a Mg metal anode and an anode-free battery with a MXene film as an anode current collector, as well as schematic diagrams of the porous Mg deposition on Cu and the horizontal Mg deposition behavior on Ti_3_C_2_T_x_ MXene film. (**h**) (**i**,**ii**) are the plating/stripping performances of Mg/Cu and Mg/Ti_3_C_2_T_x_ batteries at 1.0 mA/cm^2^ and 50% DOD, respectively. (**i**) Cycling performance test of an anode-free battery with an activated Ti_3_C_2_T_x_ MXene film. (**g**–**i**) Reproduced with permission [161]. Copyright 2023, Wiley-VCH. (**j**) Comparison of rate performances of mesoporous Mn_2_O_3_ and mesoporous Mn_2_O_3_@TiO_2_@MXene. (**k**) Comparison of rate performances of mesoporous Mn_2_O_3_ and mesoporous Mn_2_O_3_@TiO_2_@MXene. (**j**,**k**) Reproduced with permission [160]. Copyright 2023, Wiley-VCH. (**l**) Zeta potentials of different Ti_3_C_2_T_x_-based materials. (**m**) Cycling performances of different Ti_3_C_2_T_x_-based batteries. (**l**,**m**) Reproduced with permission [162]. Copyright 2024, Wiley-VCH.

The MXene surface is rich in end groups such as -OH, -O, and -F, which regulate the adsorption and desorption behavior of ions through electrostatic interactions or hydrogen bonding. For example, the -OH and -O groups enhance the hydrophilicity of MXene and facilitate ion transport in aqueous electrolytes. And the -F group regulates the electronic structure and optimizes the desolvation process of ions. These embedded ions are first adsorbed on the surface and then diffuse toward the interlayer of MXene, forming a double ionic layer within a monolayer sharing the same sites [154]. As embedding/de-embedding proceeds, the interlayer spacing widens due to solvent molecules and trapped cation column support. Notably, in aqueous zinc–ion batteries, Zn^2+^ binds to water molecules to form the hydrated ion [Zn(H_2_O)_6_]^2+^, whose effective diameter significantly expands from 0.15 nm for bare ions to 0.86 nm. And it will hinder the diffusion of ions in the narrow space of MXene, leading to capacity loss. To address the performance degradation caused by narrow interlayer spacing, Zhao et al. [155] employed a 3D macroporous pre-intercalated pure Mg_0.21_Ti_3_C_2_T_x_ MXene-based film as the cathode for multivalent ion batteries (MIBs). However, pure MXenes are prone to interlayer restacking during use due to van der Waals forces and mechanical stress generated during cycling. Additionally, they exhibit significant volume changes during ion intercalation, posing challenges to their structural stability. Peng et al. [156] employed diamines of different sizes as pillars, which were inserted into MXene interlayers via a one-step amination reaction and covalently crosslinked with surface (-OH/-O) functional groups. This approach enabled precise regulation of interlayer spacing and formation of three-dimensional (3D) porous hydrogel/aerogel structures, simultaneously enhancing specific capacitance and effectively suppressing structural collapse during long-term cycling. Moreover, the surface functional groups of pure MXene batteries and the reversible capacity of their double-layer capacitive structure are lower than those of conventional zinc–ion batteries. Therefore, the direct use of pure MXenes as electrode materials remains a challenge. Generally, during crystal nucleation and growth, a large amount of two-dimensional (2D) conductive materials such as MXenes are introduced as conductive substrates for in situ growth to construct heterostructures [157,158]. Introducing a certain amount of single-layer Ti_3_C_2_ MXene thin nanosheets to regulate the growth structure of vanadium oxide crystals V_2_O_5_·6H_2_O (HVO) significantly enhances electrochemical performance by introducing defects into the cathode material. Scanning electron microscopy (SEM) observation (Figure 13a) reveals that HVO is selectively grown at specific sites on Ti_3_C_2_ MXene, causing its morphology to transition from a multi-layer self-stacked wrinkled structure to a reticular porous structure (T-HVO). Electrochemical characterization of the obtained cathode material is shown in Figure 13d. When the current density increases from 0.2 to 30 A g^−1^, the specific capacity of the T-HVO-20 h electrode at different current densities is higher than that of the pure HVO electrode without MXene. Additionally, after 1000 cycles, the T-HVO electrode still maintains a reversible capacity of 276.9 mAh g^−1^. In contrast, the pure HVO electrode without MXene has an initial capacity of 336.9 mAh g^−1^, but its capacity retention drops to only 48 mAh g^−1^ after 1000 cycles. In addition, in the Ragone diagram (Figure 13b), the T-HVO electrode exhibits a remarkable energy density of 309.2 Wh/kg and a power capacity of 17.6 kW/kg, which are far higher than those of the HVO electrode. The significant improvement in electrochemical performance can be attributed to the following reasons. The O functional groups with low binding energy on the MXene surface form V-O-Ti bonds with the vanadium oxide crystals, resulting in a strong interfacial interaction between the two phases. Under the induction of MXene, a large number of bicoordinate oxygen defects are generated at the lattice interface. Along with the increase in the EPR intensity signal, the number of unpaired electrons increases, causing more V^5+^ to be transformed into V^4+^ through electron transfer. As shown in Figure 13c, with the extension of the induction growth time, both pairs of oxidation peaks shift to the left (the blue part represents V^5+^/V^4+^, and the red part represents V^4+^/V^3+^). However, the area of the first pair of redox peaks corresponding to V^4+^/V^3+^ expands significantly, indicating that the generation of oxygen defects leads to an increase in the proportion of V^4+^. It should be noted that these bicoordinate oxygen defects mainly enhance the reactivity of the V^4+^/V^3+^ reaction, and promote the transfer of charges and the adjustment of active sites. Similarly, in the T-HVO electrode, the current response in Zone II broadens (Figure 13e), proving that the redox reaction of V^4+^/V^3+^ is significantly improved. It also demonstrates that the existence of oxygen defects does improve the reactivity, and increase the solid diffusion rate of zinc ions and the conductivity of the electrode, which is beneficial for accelerating the electrochemical intercalation reaction and significantly improving the capacity and rate performance of the zinc–ion battery.

Magnesium–ion batteries (MIBs), also known as multivalent ion batteries, are considered to be one of the next generation of low-cost, high-energy energy storage devices [163]. MIBs have a larger specific capacity than traditional LIB batteries [164]. However, unlike the SEI film which is beneficial to the performance of lithium–ion batteries, magnesium–ion batteries mostly use organic or ionic liquid electrolytes, and Mg^2+^ has strong electrostatic interaction and low conductivity. The formed solid electrolyte film is not conducive to the reversible insertion/extraction and interfacial diffusion of ion fluxes on the cathode, and even causes the inactivation of the electrode surface, resulting in capacity attenuation [165]. Through chemical adsorption by the oxygen (-O) and fluorine (-F) terminations on the MXene (Ti_3_C_2_T_x_) surface, the local Mg^2+^ concentration is significantly enhanced, addressing the migration barrier caused by the strong Coulombic force of multivalent ions. Li et al. [160] reported a three-phase heterogeneous structure of mesoporous Mn_2_O_3_@TiO_2_@MXene, and its morphological characterization is shown in Figure 13f. It has an initial capacity as high as 241.5 mAh g^−1^ at 0.1 A g^−1^ and exhibits excellent electrochemical rate performance (Figure 13k). When an aqueous magnesium–ion capacitor composed of mesoporous Mn_2_O_3_@TiO_2_@MXene as the positive electrode material and activated carbon (AC) as the negative electrode material was subjected to 2400 cycles of testing at a current density of 1.0 A g^−1^, its capacity retention rate was still 99.91% (Figure 13j). The heterogeneous structure formed by MXene and TiO_2_ particles derived from Mn_2_O_3_ can mitigate the volume expansion of manganese sesquioxide, significantly solving the problems of interface separation and decline in electrochemical performance caused by the instability of other heterogeneous structures. Apart from the acceleration of magnesium–ion diffusion by the inherent electric field generated by the heterojunction, the improvement in performance is mainly due to the large number of interfaces (specific surface area of 37.73 m^2^/g) generated by the three-phase heterogeneous structure of mesoporous Mn_2_O_3_@TiO_2_@MXene, which can provide abundant active sites for magnesium ions.

In addition, apart from the problem of the rate of movement, insertion, and extraction of magnesium ions in the electrolyte, how to design a reasonable electrode structure to avoid the waste of active metals caused by using large pieces of active metals as anode electrodes and to reasonably increase the energy density of the battery is also an important issue [166]. Based on this, researchers have designed an anode-free battery structure. For example, in rechargeable magnesium–ion batteries, an inactive material is used to construct a current collector. During the initial charging and discharging cycles, Mg^2+^ is deposited on it, and it participates in the electrode reaction as the negative electrode in subsequent cycles [167]. The cathode rich in magnesium ions is the main source for magnesium deposition on the anode during charging, thus reducing the volume and weight of the battery and avoiding the decrease in energy density caused by large anode materials [168]. However, how to ensure that magnesium can be continuously deposited during the continuous charging process, maintain a stable electrode structure, and suppress the growth of dendrites during deposition remains an issue to be resolved for current anode-free magnesium metal batteries.

Li et al. [161] prepared an anode-free magnesium metal battery by parallel stacking single-layer Ti_3_C_2_T_x_ films as current collectors. The schematic diagrams of a standard battery with a Mg metal anode, an anode-free battery with Ti_3_C_2_T_x_ films as anode current collectors, and the porous Mg deposition behavior on the corresponding traditional Cu current collector as well as the horizontal Mg deposition on the surface of the Ti_3_C_2_T_x_ current collector are shown in Figure 13g. Due to the unique two-dimensional layered structure and excellent mechanical properties of Ti_3_C_2_T_x_, it can improve the adhesion of magnesium metal during the initial deposition and control the volume change of the electrode. Moreover, the surface of the Ti_3_C_2_T_x_ film is uniformly distributed with functional groups such as magnesiophilic active oxygen and active fluorine, which attract magnesium ions and form a dense MgF_2_-rich SEI film around the inactive current collector anode. In addition, Ti_3_C_2_T_x_ has an approximate lattice matching degree (≈96%) with Mg, thereby being able to guide the nucleation and growth of Mg on the surface of MXene sheets. Working together with the uniformly high magnesium–ion concentration around the SEI film, it results in a uniform and stable horizontal electrodeposition of Mg. The results show that, as shown in Figure 13h(ii), when the battery is cycled at a current density of 1.0 mA/cm^2^ with a fixed capacity of 1.0 mAh/cm^2^ (QC) until the charging voltage reaches the limit of 1 V (QS), the Ti_3_C_2_T_x_ MXene film anode with secondary deposited magnesium metal after pretreatment can still maintain a Coulomb efficiency (CE) of 99.4% and a high magnesium utilization rate (depth of discharge, DOD) of 50% after 364 cycles of testing at a high current density of 5.0 mA/cm^2^. Compared with the uneven magnesium deposition produced when the traditional copper foil is used as the anode current collector (Figure 13h(ii)) (70 cycles, CE = 98.4%), the charging curve of Ti_3_C_2_T_x_ MXene as an inactive anode is more stable (Figure 13h(i)), achieving unprecedented high efficiency and long-life magnesium plating/stripping performance. In addition, a full battery was composed of Ti_3_C_2_T_x_ MXene as an anode current collector and a coupled pre-magnesified Mo_6_S_8_ cathode, and its cycling performance was tested at a current density of 0.1 C (1C = 128 mA g^−1^) (Figure 13i). The initial discharge capacity of the battery was 63.2 mAh g^−1^, and after 100 cycles, it still had a capacity retention rate of 47.2%, which is much higher than that of the traditional standard magnesium metal anode battery, showing good cycling stability and relatively high energy density.

In addition to zinc–ion and magnesium–ion batteries, more expensive aluminum–ion batteries have extremely rich reserves in the earth’s crust, and trivalent aluminum can carry more electrons, with a higher theoretical capacity (2980 mAh g^−1^) and energy density [169,170,171]. But the problems are also very prominent. First, similar to magnesium–ion batteries, the migration of ions and electrons is hindered by the electrolyte as well as by stronger electrostatic interactions [172]. Second, the embedding/de-embedding process of aluminum ions involves the migration of bulk AlCl^4–^ rather than Al^3+^, which leads to a further decrease in the diffusion rate as well as a further increase in the electrode material requirements. Guan et al. [162] proposed a strategy to regulate the surface charge of the MXene cathode to overcome the above difficulties. Although using pure MXene as the cathode material, the insertion of a large number of AlCl^4−^ ions during the charging and discharging process can expand the layer spacing of the neighboring MXene layers, which can effectively solve the problems of stacking and volume expansion between the layers. However, the Ti_3_C_2_T_x_ MXene obtained after etching with a large number of surface functional groups such as OH and COOH will make it negatively charged as a whole, which further causes a certain electrostatic repulsion to the embedding/de-embedding process of AlCl^4−^. The zeta potential of Ti_3_CNT_x_ (13.4 mV) was found to be much lower than that of Ti_3_C_2_T_x_ (30.4 mV) by doping N with Co atoms, as shown in Figure 13l. The zeta potential of Ti_3_CNT_x_ (13.4 mV) was found to be much lower than that of Ti_3_C_2_T_x_ (30.4 mV) by doping N with Co atoms. It indicates that the doping of N effectively reduces the surface negative charge density, while the addition of Co causes the surface charge to finally become positive. This change in surface charge reverses the electrostatic interaction between MXene and active anions from electrostatic repulsion to electrostatic attraction, allowing more AlCl^4−^ ions to be embedded in the interlayer of Ti_3_CNT_x_@Co, thus increasing its capacity. The results show that the Ti_3_CNT_x_@Co cathode–Al anode cell has an ultra-high capacity of up to 240 mAh g^−1^ compared with the graphite cathode used in the past (Figure 13m), and still maintains a high reversible capacity after 600 cycles.

The application of MXenes in multivalent ion batteries (MVIBs) essentially leverages their 2D layered structure, tunable surface chemistry, high electrical conductivity, and other multifunctional properties to construct integrated systems. Existing studies have effectively alleviated challenges such as slow multivalent ion migration, high interfacial impedance, and dendrite growth through strategies like interlayer spacing regulation, heterostructure construction, and surface charge engineering. However, further exploration is still needed in areas such as interfacial chemistry, multiscale structural design, and material compatibility. Future efforts could involve combining high-throughput calculations to screen optimal functional group combinations or developing MXene-based composite electrolytes, aiming to promote MVIBs as complementary and potentially superior alternatives to lithium–ion batteries.

## 5. Conclusions and Outlook

MXene is an emerging 2D layered material, which has been widely used in energy storage in recent years due to its unique properties. This paper systematically introduces the structure, properties, and general synthesis method of MXene. It focuses on the great potential of MXene for applications in the field of lithium–ion, sodium–ion, lithium–sulfur, and multivalent ion batteries. The problems faced by different energy storage schemes and the efficient and safe solutions proposed for these problems are studied and outlined. Summarizing the research in recent years, it can be found that the indirect use of MXene is primarily aimed at enhancing electrode performance through the following aspects.

1. Utilizing its excellent conductivity and two-dimensional layering, it is used as a conductive substrate for composite electrodes. MXene’s rich surface functional groups grafting/growth of other electrochemically active phases such as transition metal oxides/sulfides or even a variety of common composite composite composition of different dimensions of the conductive framework. The linkage between the two is generally achieved by direct chemical synthesis through physical mixing (mechanical ball-milling mixing or wet dispersion freeze-drying mixing), in situ growth or solvothermal methods, or by electrostatic/other forces to realize the self-assembly of MXene and the anode material into a solid transition layer or to form heterostructures connected by covalent bonds. The electrochemically active phase is able to provide higher capacity storage sites by virtue of its special electronic structure and variable valence state of the chemistry, thus enabling efficient charge transfer in electrochemistry.

2. Design of the structure of the electrode material. Early studies have shown that the use of MXene to build three-dimensional structures such as three-dimensional skeleton, aerogel, etc., as the cathode framework and grafting composite other materials with excellent electrochemical properties, not only can retain the characteristics of the electrochemically active phase of the high-capacity, but also use the high electrical conductivity and mechanical stability of MXene to avoid the cathode volume expansion brought about by the problem of pulverization. It is also very important for the stable output of the battery under long cycle.

3. Utilizing the special layer structure of MXene. How to avoid stacking between the layers and expand the layer spacing, which can further accommodate larger and more charged multivalent ions, and also bring larger specific surface area, which are very important for the capacity of the battery, reducing the formation of dendrites, and enhancing the kinetics of ion embedding/de-embedding to the cathode.

4. In the above study, it can also be found that the modulation of MXene surface functional groups into active sites with electrostatic attraction to multivalent ions also contributes significantly to the battery capacity enhancement.

The charging and discharging process of multivalent ion batteries is very complex, with both mass transfer problems of ions in specific electrolytes, desolvation at the electrode interfaces and diffusion problems of reduction and deposition on SEI membranes, which often lead to slow ion kinetics and are difficult to replicate again by conventional single methods. However, high-priced ion batteries have the potential for greater current density, faster impulse discharge, and are currently one of the best alternatives to replace lithium–ion batteries as a new and efficient energy storage device of the era. Therefore, the current research needs to fully consider the excellent performance and special structure of MXene itself for a comprehensive electrode design in order to avoid material wastage and utilize its best performance. This review aims to consolidate the past research results and summarize the latest research results in recent years, so as to catalyze the generation of new MXene-based electrode materials with even better performance.

Although MXene-based electrode materials have been spread in various fields of rechargeable batteries and have made great progress, there is still a lot of room for development for rechargeable batteries through the advancement of research and the development and innovation of a steady stream of new materials.

1. The complex preparation process is one of the key factors limiting the widespread development of MXene. Despite the toxicity of hydrofluoric acid (HF) and the controversies over its environmental hazards, which make large-scale production outside laboratory settings difficult, alternative etching methods such as alkaline etching, electrochemical etching, molten salt etching, and chemical vapor deposition (CVD) remain in the early stages of research. These methods involve multiple reaction and etching steps, which also increase production costs and reduce efficiency in practical applications. Therefore, as the only synthesis method that currently balances high delamination efficiency and low defect density, HF etching to prepare Ti_3_C_2_T_x_ MXene remains the standard for producing high-quality MXenes at the laboratory stage. In the future, efforts should be dedicated to developing greener fluoride-free, simpler, and more efficient synthesis methods. Such methods should enable precise control over MXene sheet thickness, tuning of surface functional group types, and adjustable interlayer spacing.

2. The specific mechanism of MXene in electrodes still has a great deal of room for research, and the effects of the type of MXene, the number of layers, as well as the type and number of functional groups on the performance of composite anodes are not yet known. The future should be devoted to the study of the connection between MXenes and other active phases to explore the physicochemical principles behind the electrochemical performance enhancement.

3. Bimetallic MXenes (such as Ti_2_NbC_2_T_x_) enable directional regulation of electronic structure and chemical bond properties by introducing a second transition metal. Studies have shown that Nb incorporation significantly expands the interlayer spacing of Ti_2_NbC_2_T_x_ (from 1.05 nm to 1.25 nm), providing efficient diffusion channels for multivalent ions like Zn^2+^ and Mg^2+^ (with diffusion rates increased by over 50%). This effectively mitigates the kinetic limitations of ion transport in traditional MXenes caused by narrow interlayer spacing. Additionally, the compositional tunability of bimetallic MXenes (Ti/V, Nb/Mo combinations) induces charge redistribution, forming local electron-rich regions that activate more ion adsorption sites. Through precise design of transition metal composition and interfacial chemical states, directional optimization of the material’s intrinsic properties is achieved, offering new insights for developing universal energy storage materials. However, most current electrode materials still use single-metal MXenes. Therefore, future research should further investigate the synthesis, applications, and energy storage mechanisms of bimetallic MXenes in batteries.

## Figures and Tables

**Table 1:** Current methods of preparing MXene and the differences between the different routes.

Synthesis Methods	Characteristics	Advantages	Disadvantages
HF Etching	The A layer in the MAX phase was etched using F^−^ with Al^3+^ to generate MXene containing -F/-OH/-O end groups.	The process is simple, high yield, low cost, and universally applicable.	HF is toxic and harmful to the environment; difficult to peel off in a single layer and easy to build up.
In situ generation HF etching	Fluorine salts (LiF/NH_4_F) were mixed with acid to generate HF in situ to etch the A layer atoms.	Much gentler than direct HF etching for large nanosheets (lateral dimensions > 1 μm).	Fluorine-containing reagents are still needed, and the yield is lower than that of HF etching.
Alkali etching	High-temperature strong bases (NaOH/TMAOH) dissolve the Al layer to produce MXene, which is dominated by -OH end groups.	Fluorine-free; excellent hydrophilicity, layer spacing can be controlled by cationic intercalation.	Harsh reaction conditions.
Electrochemical etching	Applying a voltage selectively etches the A layer to produce MXene with a -OH dominated surface.	Fluorine-free; precise etch depth control for low-layer MXene preparation; high purity (>95%).	Complex equipment; slow etching rate; easily over-etched.
Molten salt etching	The high-temperature molten salt displaces the A layer in the MAX phase to produce MXene with end groups such as -Cl on the surface.	Fluorine-free; suitable for Si-based/nitride MAX phase etching; adjustable endbase.	High-temperature conditions; difficulty in regulating layer spacing.
Chemical deposition (CVD)	Direct synthesis of MXene by vapor phase deposition without MAX phase.	Nitride MXene can be prepared; precise control of size/thickness and few surface defects.	The high cost of equipment makes large-scale production difficult.

## Data Availability

No new data were created.
